# Nickel Hyperaccumulator Biochar Sorbs Ni(II) from Water and Wastewater to Create an Enhanced Bio-ore

**DOI:** 10.1021/acsenvironau.2c00028

**Published:** 2022-09-16

**Authors:** Rachel A. Smoak, Jerald L. Schnoor

**Affiliations:** ‡ Department of Civil and Environmental Engineering, University of Iowa, 4105 Seamans Center for the Engineering Arts and Sciences, Iowa City, Iowa 52242, United States; § IIHR − Hydroscience and Engineering, University of Iowa, 100 C. Maxwell Stanley Hydraulics Laboratory, Iowa City, Iowa, 52242, United States

**Keywords:** Odontarrhena chalcidica, Alyssum murale, hyperaccumulator, biochar, nickel, nickel sorption, electroplating wastewater

## Abstract

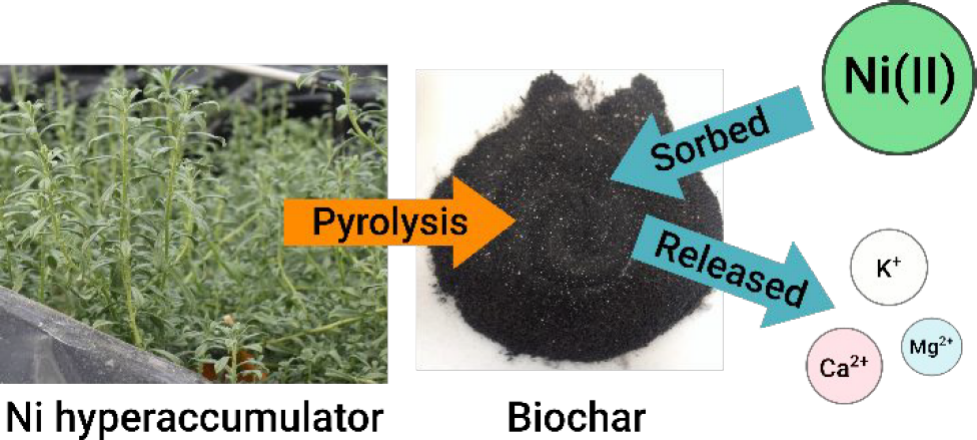

Nickel (Ni) hyperaccumulators make up the largest proportion of hyperaccumulator plant species; however, very few biochar studies with hyperaccumulator feedstock have examined them. This research addresses two major hypotheses: (1) Biochar synthesized from the Ni hyperaccumulator *Odontarrhena chalcidica* grown on natural, metal-rich soil is an effective Ni sorbent due to the plant’s ability to bioaccumulate soluble and exchangeable cations; and (2) such biochar can sorb high concentrations of Ni from complex solutions. We found that *O. chalcidica* grew on sandy, nutrient-poor soil from a Minnesota mining district but did not hyperaccumulate Ni. Biochar prepared from *O. chalcidica* biomass at a pyrolysis temperature of 900 °C sorbed up to 154 mg g^–1^ of Ni from solution, which is competitive with the highest-performing Ni sorbents in recent literature and the highest of any unmodified, plant-based biochar material reported in the literature. Precipitation, cation exchange, and adsorption mechanisms contributed to removal. Ni was effectively removed from acidic solutions with initial pH > 2 within 30 min. *O. chalcidica* biochar also removed Ni(II) from a simulated Ni electroplating rinsewater solution. Together, these results provide evidence for *O. chalcidica* biochar as an attractive material for simultaneously treating high-Ni wastewater and forming an enhanced Ni bio-ore.

## Introduction

Biochar is produced by heating carbonaceous materials in an oxygen-poor environment, resulting in biogas, bio-oil, and biochar products.^1^ Biochar’s proposed uses are extremely varied in scope and application. Biochar is a carbon capture technology and a soil amendment known to increase crop yields on marginal lands.^1,2^ Biochar is also used to remove toxic organic and inorganic pollutants from water; to produce energy storage devices; and in carbon-based composites that are components of plastics, cements, and ceramics.^3,4^

The properties of a specific biochar influence its uses. For example, sorption of inorganic pollutants by biochar in water treatment often relies on cation exchange capacity, electrostatic attraction conferred by π bonds in the biochar, adsorption to metal-binding surface sites, and precipitation of metal pollutants at high pH.^5^ Although the process for making biochar is conceptually straightforward, many factors influence the final properties of the biochar. Treatment temperature influences all physical properties of the biochar, and higher pyrolysis temperature increases alkalinity, cation exchange capacity, and surface area while decreasing the concentration of surface functional groups and altering mineral phases.^5,6^ Activation through carbonization after contact with acidic solution, basic solution, or steam alters pore structure, surface area, pH, and surface functional groups.^7,8^ However, the most fundamental factor that influences biochar properties is the feedstock.^9^ Feedstock properties influence specific surface area, cation exchange capacity, and nutrient availability; biochar properties are often dependent on the feedstock’s structure and elemental content.^10^

Metal hyperaccumulating plants are species that grow on metalliferous soils and accumulate high amounts of metal in their aerial portions without suffering toxic effects; the concentration of metal required to be considered a hyperaccumulator varies by element.^11^ Hyperaccumulator biochars possess unique properties due to the reactivity of their high metal contents during pyrolysis. For example, hyperaccumulated metals are known to change the hemicellulose reaction pathway during microwave pyrolysis, catalyze H_2_ production during pyrolysis, and increase sorption of heavy metals to the resultant biochar.^12−14^ Recent work has primarily delved into using As, Cd–Zn, Mn, and Zn hyperaccumulators as biochar feedstocks with the goals of heavy metal sorption, catalysis, and bio-oil production for power generation and chemical synthesis.^12,14,15^ However, the vast majority of known hyperaccumulators are Ni hyperaccumulators.^16^ To the best of our knowledge, only two studies have focused on biochar production from Ni hyperaccumulator biomass: Doroshenko et al. working with *Stackhousia tryonii* F.M. Bailey and *Odontarrhena bertolonii* (Desv.) L.Cecchi & Selvi who found that hyperaccumulated Ni led to increased biochar production and our group working with *Odontarrhena chalcidica* (Janka) Španiel, Al-Shehbaz, D.A.German & Marhold (formerly known as *Alyssum murale*).^13,17^

*O. chalcidica* is a perennial Ni hyperaccumulator native to ultramafic soils in the Mediterranean region that is known to hyperaccumulate Ni from soils with sufficient phytoavailable Ni even when planted outside of its native range.^18,19^ It is known to selectively accumulate up to 30% Ca by dry weight in leaf trichomes, primarily as a carbonate or oxalate, and to accumulate Ca from soils with low Ca concentrations, which could increase the alkalinity and cation exchange capacity of any biochar made from the plant.^20,21^ Due to its high Ni accumulation and ability to be cropped in many temperate climate zones, *O. chalcidica* is a primary species being considered for use in the nascent agromining industry.^22^ Agromining, or “farming for metals”, uses hyperaccumulator plants to concentrate metals from soil, harvests the plants, ashes the biomass to make bio-ore, and extracts the metals to make metal or metal products.^23^ Our group’s previous work focused on synthesizing an enhanced bio-ore by pyrolyzing *O. chalcidica* biomass into biochar and sorbing aqueous Ni from solution to it.^17^ The previous biochar study demonstrated high Ni sorption capacity, especially in biochar formed at high pyrolysis temperature. This indicated that *O. chalcidica* biochar could be processed into an enhanced bio-ore by sorbing Ni from mining-impacted water, industrial wastewater, and other high-metal water sources.^24−26^ This was, to the best of our knowledge, the first demonstration of a value-added product created from the direct pyrolysis of Ni hyperaccumulator biomass. However, the biomass was grown on Ni-spiked potting soil, not natural soil, and only Ni sorption capacities were explored. This work investigates the sorption characteristics of *O. chalcidica* biochar pyrolyzed at high temperature and the sorption of Ni(II) from industrially relevant solutions to form an enhanced bio-ore.

To determine the utility of *O. chalcidica* grown on natural soil as a biochar sorbent feedstock, we grew the plants on soil from a northeastern Minnesota mining zone; pyrolyzed the biomass at 900 °C; tested the sorption capacity, pH dependence, and sorption kinetics of the biochar for Ni(II); and measured its capacity to remove Ni(II) from synthetic high-Ni extraction and wastewater solutions.

## Experimental Section

Additional information on all experimental procedures can be found in the data deposit associated with this work.^27^ All data analysis was performed in R, and figures were constructed in R using the package “ggplot2”.^28,29^

### Soil Collection and Characterization

Candidate soils were identified in northeastern Minnesota (MN) using predictions of Ni concentration in the A horizon and areas with high reported Ni concentrations in mineral concentrate, silt, clay, and humus samples.^30−32^ A Short-Term Geological Authorization was secured from the MN Department of Natural Resources to survey Ni soil concentrations in prescribed areas and collect up to 380 L for experimental use; rocks > 10 cm in diameter were left in the field. After returning the soil to the laboratory, it was air-dried and homogenized through coning and quartering.^33^ Promix potting mix was used as a control soil in this work. Subsamples of the MN soil were sent to Minnesota Valley Testing Laboratories, Inc. for characterization and analysis.

The total metal contents of the soils were measured by portable X-ray fluorescence spectroscopy (pXRF, INNOV-X Delta Premium XRF, Olympus Corporation, Tokyo, Japan) and the bioavailable metal contents were approximated by parallel HCl and CaCl_2_ extractions of soil subsamples analyzed by inductively coupled plasma-optical emission spectrometry (ICP-OES, Varian 720-ES, Agilent Technologies, Santa Clara, CA) before and after the experiment.^34−36^

### Plant Growth, Characterization, and Mixing

After homogenization, the MN soil was divided into five 114 L plastic grow bags, and potting mix was placed into five additional grow bags. Each grow bag contained approximately 75 L of soil or potting mix. All bags were located in a greenhouse in Coralville, Iowa. *O. chalcidica* “Kotodesh” seeds (Albania, 1998) obtained from the U. S. Department of Agriculture (Beltsville, MD) were sprouted in free-draining nursery trays in a laboratory plant growth chamber. Forty healthy plants were transplanted to the 10 grow bags. The plants were watered by an automatic watering system; the watering rates were adjusted and grow bags were irrigated with additional water as necessary. A sample of the greenhouse irrigation water was microwave digested (ETHOS UP, Milestone Srl, Sorisole, Bergamo, Italy) with HNO_3_ and HCl according to EPA Method 3015A and analyzed for metal content by ICP-OES.^37^ The plants were supplemented with Miracle-Gro Water-Soluble All Purpose Plant Food according to manufacturer instructions: 2.5 mL of Miracle-Gro was mixed into 3.8 L of water and applied every 2 weeks to each grow bag, delivering approximately 0.5 g of total N, 0.2 g of P_2_O_5_, and 0.3 g of K_2_O per fertilization. The full chemical components of the Miracle-Gro are described in Table S1. The plants were allowed to grow for approximately 9 months (March to November 2020), and the aerial portions were harvested before the plants entered winter dormancy.

The harvested plant material was triple washed with DI water, dried, and ground. Individual plant metal measurements were made with pXRF, and differences in composition were examined through a *k*-means clustering analysis. Because the plants showed limited interplant variation when grown on the same media, plants were grouped into “MN plant” and “C plant” master mixes, which contained plants grown on Minnesota and control potting soils, respectively. pXRF and ICP-OES analyses were employed to measure the metal concentration of the master mixes. The preprogrammed microwave digestion procedure using HNO_3_ and H_2_O_2_ (SK-AGRICULTURE-004) was used to prepare samples for ICP-OES analysis.^38^

### Biochar Synthesis and Characterization

The MN plant and C plant master mixes were pyrolyzed at 900 °C into the biochars MN900 and C900, respectively. Batches of ∼20 g of biomass were loaded into each of four refractory ceramic evaporating dishes, placed in the heated zone of a tube furnace (OTF-1200X, MTI Corporation, Richmond, CA) inside a fused quartz tube, and purged with nitrogen gas (N_2_). The N_2_ flow rate was adjusted to ∼70 standard cubic centimeters per minute (sccm), and the furnace was ramped to 900 °C at 5 °C min^–1^, held there for 90 min, and allowed to cool naturally under N_2_ until 160 °C. Due to furnace batch size limitations, this procedure was repeated for the available plant biomass, and the biochar batches made from the same plant master mix were combined.

The two biochars were characterized by pXRF and microwave digestion for ICP-OES measurement according to EPA Method 3051A.^39^ ICP-OES analysis was conducted on filtered and acidified samples for Ca, Cd, Cu, Fe, Mg, Mn, Ni, K, Sr, Ti, and Zn with a 1 mg L^–1^ Y internal standard added at a 1:1 volumetric ratio upon injection to the instrument. The pH of each biochar was measured in DI water at a 1:200 biochar:water mass ratio. The acid neutralizing capacity of the biochars was determined by repeated addition of 0.05 M HCl until the biochars reached pH 7, as described in the literature.^40^ The neutralized biochars were then used to measure cation exchange capacity at pH 7 by the exchangeable base cation method with ICP-OES quantification and the ammonium displacement method quantified spectrophotometrically by the ammonium salicylate method.^40,41^ Multipoint Brunauer–Emmett–Teller (BET) analysis (Quantachrome Instruments 4200e, Anton Paar, Boynton Beach, FL), sorption/desorption isotherm analysis, and scanning electron microscopy (SEM, S-3400N, Hitachi, Ltd., Tokyo, Japan) were used to characterize the biochar surface. Biochar SEM analysis was conducted after adhering a small strip of folded copper tape to an SEM stub and pressing a thin layer of biochar to the tape.

### Biochar Sorption Isotherms

In order to determine the Ni(II) sorption capacity of biochar synthesized from *O. chalcidica*, batch sorption isotherm experiments were performed. Dilutions of 10 and 20 mM Ni(II) stock were prepared in 10 mM NaNO_3_ such that the approximate Ni(II) concentration ranged between 0 and 20 mM Ni, and the pH of each was adjusted to approximately pH 5. Duplicate combinations of each biochar (50 mg)-Ni(II) dilution (10 mL) and negative controls with no biochar were prepared in 20 mL glass vials. The vials were shaken for 24 h, the supernatant was syringe filtered (0.45 μm), and aliquots were acidified with 4% HNO_3_ for ICP-OES analysis and stored in the dark at 4 °C prior to pH measurement and ICP-OES analysis. The remaining biochar in the vials was dried at 105 °C overnight and stored at room temperature.

ICP-OES analysis was conducted on acidified samples for Ca, Cd, Cu, Fe, Mg, Mn, Ni, K, Sr, Ti, and Zn with a 1 mg L^–1^ Y internal standard based on metal presences indicated by pXRF measurements. Calibration curves were recalculated every 10 samples, and solutions were diluted so that identified elements were within the range of the calibration curve. The Y internal standard was added by the instrument immediately preceding injection and used for normalization between measurements. The limits of detection (LOD) were measured according to EPA Method 6010D as three times the standard deviation of 10 blank samples, and LODs for different procedures were backcalculated using the specific sample dilutions.^42^

The specific metal removal and percent of metal removed by each biochar sample were calculated as in our previous paper.^17^ Adsorption data were fitted using the two-parameter Freundlich and Langmuir isotherms as well as the three-parameter Redlich-Peterson isotherm.^43^ All three models were evaluated nonlinearly in R using the PUMPAIM package.^44^

### Biochar Sorption Kinetics

To evaluate the sorption kinetics of Ni(II) to *O. chalcidica* biochar, Ni was analyzed in the aqueous phase of biochar-solution systems at multiple time points. A volume of 200 mL of 5 mM Ni(II) in 10 mM NaNO_3_, pH 5 solution was mixed with 1 g of biochar in a closed serum bottle, and aliquots were removed by syringe at 1, 5, 10, 15, 30, 60, 300, and 1440 min (1, 5, and 24 h).^45^ Each experiment was conducted in duplicate, and two control samples with no biochar underwent the same procedure. The filtering, acidification, and ICP-OES analysis procedures followed the method described for the sorption isotherms.

### Biochar Sorption pH Dependence

To determine the effect of pH on *O. chalcidica* biochar’s Ni(II) sorption capacity, biochar samples (50 mg) were contacted with 5 mM Ni(II) in 10 mM NaNO_3_ solution of pH 0.5–11 (10 mL), and the final pH and solution Ni concentrations were measured as described above. Each experiment, including controls with no biochar, was conducted in duplicate. The filtering, acidification, and ICP-OES analysis procedures followed the method described for the sorption isotherms. The Ni speciation was also calculated for the experimental system at pH values between 0.5 and 12 using the software MINEQL+ 5.0 (Environmental Research Software, Hallowell, ME).^46^

### Biochar Sorption from Complex Mixtures

To determine the ability of *O. chalcidica* biochar to remove Ni(II) from complex mixtures, sorption was measured in simulated *O. chalcidica* leachate and simulated Watts bath Ni electroplating rinsewater. The simulated *O. chalcidica* leachate was an aqueous mixture of metals and low molecular weight carboxylic acids that was prepared based on the composition of elements extracted from dried, ground *O. chalcidica* biomass grown on native serpentine soil with water by Guilpain et al., and included K^+^, Ni(II), Mg(II), Ca^2+^, and Fe(III) primarily as nitrates (Table S2).^47^ A simulated leachate metals mixture, which lacked the organic acids, was also tested. Both were adjusted to pH 5.7 using concentrated KOH; 50 mg biochar were used in these experiments. The simulated Ni electroplating rinsewater composition and pH were based on effluent from the common Watts Ni bath electroplating solution and included NiCl_2_, NiSO_4_, and H_3_BO_3_ (Table S2).^48^ The pH was adjusted to 4.2 using concentrated HCl and KOH as needed, and 0.2 g biochar were used in these experiments. Trials were conducted with all biochars and biochar-free controls in duplicate. The filtering, acidification, and ICP-OES analysis procedures followed the method described for the sorption isotherms. The Ni speciation was calculated for the Ni electroplating rinsewater system at pH values between 0.5 and 12 using MINEQL+ 5.0; a similar speciation calculation is available in the literature for the *O. chalcidica* leachate system.^46,47^

### Biochar Postsorption Characterization

Selected biochar samples were analyzed after the sorption experiment to determine elemental content, changes in surface structures, elemental distribution, and crystalline phases present. Metal concentrations in the biochar after 24 h of contact with the 10 mM Ni sorption isotherm solution were determined by microwave digestion and ICP-OES as previously described for biochar. Changes in surface structures and elemental distribution were determined using combined SEM and energy dispersive X-ray spectroscopy (EDS, QUANTAX EDS, Bruker Corporation, Billerica, MA) methods. Crystalline phase presence was determined through X-ray diffraction (XRD) performed on duplicate-pooled biochar after 24 h of contact with the 5 mM Ni sorption isotherm solution at Iowa State’s Materials Analysis and Research Laboratory (Siemens D500 Powder Diffractometer, Bruker Corporation, Billerica, MA) and analyzed using the Jade 9.5 software (Materials Data Incorporated, Livermore, CA).

## Results and Discussion

Raw data and R code used to analyze the data and produce figures can be found in the data deposit associated with this work.^27^

### Soil Collection and Characterization

After surveying several sites in the area, soil was collected from UTM NAD83 Zone 15N 596029.29, 5281443.62 (Lake County, MN) in the Bald Eagle Intrusion in the layered series of the Duluth Gabbro Complex (Figure S1). The soil pH was within the range demonstrated to support *O. chalcidica* growth (Table S3).^21,49^ The total metal pXRF results show somewhat elevated levels of Ni, Sr, Cu, Fe, Ti, and Cr, as expected for soils from the area (Table S3).^30^ Further analysis at Minnesota Valley Testing Laboratories, Inc. indicated that the collected soil was sandy (905 mg g^–1^) with very low organic matter content (7 mg g^–1^) and poor agronomic quality (Table S4). Although the concentrations of total metals were relatively high in the MN soil, the collected soil lacked the high concentrations of Ni and Co and high Mg/Ca quotient characteristic of serpentine soils; the Ti concentration was higher than typically reported in serpentine soils.^21^ The poor agronomic quality of the MN soil and low concentrations of Ni and Mg in the MN soil and potting mix likely stunted *O. chalcidica* plant growth and Ni hyperaccumulation during this experiment.^22,50^

The pH and metal content of the MN soil and potting mix were measured after the plant growth experiment. The post-experimental pXRF measurements indicated an increase in Cu and Zn concentration in both the MN soil and potting mix and an increase in Sr concentration in the potting mix following the plant-uptake portion of the experiment (Table S3). The CaCl_2_ and HCl extractable metal measurements also indicated an increase in soil concentration of Cu, Sr, Ca, Mg, and Fe in the potting mix during the plant-uptake experiment; Ca and Mg are not detectable by our pXRF method, and the Ni and Zn concentrations indicated by pXRF measurements were below the detection limit for the CaCl_2_ and HCl extractable metals methods (Table S5). The extractable metals in solution are a proxy for bioavailability to plants.^35,36^ The greenhouse water metal analysis showed that Ca, K, and Mg were present in the irrigation water (Table S6). The fertilizer applied biweekly to the plants contained K, Cu(II), and chelated Fe(III). Together, the irrigation water and fertilizer account for the elements with increased soil concentration over the course of the experiment, excluding Cu, Sr, and Zn. The irrigation water may have contained Cu, Sr, and Zn at levels below the limits of detection (LOD) of the methodology employed in this study, or the fertilizer may have been contaminated with the metals which, regardless of source, accumulated to a detectable level in the soil over the course of the experiment.

### Plant Growth and Characterization

The *O. chalcidica* plants grew on the MN soil and potting mix, although the plants grown on the potting mix grew on average twice as large. One plant grown on the potting mix died during the course of the experiment. The MN soil did not retain water well due to its high sand and low organic matter contents, and sandy soils are known to retain nutrients poorly, both of which may have contributed to the stunted growth of plants grown on the MN soil. The characteristics of the MN and potting soil were not favorable for long-term plant productivity.^51^ The pXRF measurements of the individual plants were analyzed through a *k*-means clustering analysis with nondetections of elements replaced with the LOD (Figure S2). While most plants grown on the same soil clustered together, three samples clustered with plants grown on the other soil material. Because all of the opposite-clustering samples had low amounts of biomass, they were excluded from the plant master mixes. The pXRF measurements of the MN plant and C plant master mixes detected 57 mg kg^–1^ Ni in the MN mix, a low concentration for a known Ni hyperaccumulator (Table S7). The presence of Ni in the MN plant measurement indicates that *O. chalcidica* was able to accumulate some Ni from the MN soil (pXRF bioconcentration factor = 0.77 ± 0.12) but not enough to reach hyperaccumulating levels (1000 mg kg^–1^ Ni in dry plant material), likely due to low Ni concentration in the soil compared to serpentine soils or low phytoavailability.^52^ This disqualifies similar soils in the region for use in agromining of Ni with *O. chalcidica*. The ICP-OES measurements of acid-assisted microwave digests of the plant master mixes indicate similar levels of Ca and K in all plants, and do not register the presence of any other tested metals (Table S8). Ca and K accumulated in *O. chalcidica* during the course of its growth; *O. chalcidica* is known to selectively accumulate high concentrations of Ca in its leaf trichomes.^20,53^ Both the MN and C plant mixes show similar concentrations of Ca compared to *O. chalcidica* plants grown wild in their native environments.^21^

### Biochar Synthesis and Characterization

pXRF analysis of the biochars indicates that pyrolysis increased the concentration of most metals (Table S7). The exception was Zn, which decreased in concentration after pyrolysis. Zn is known to migrate to the bio-oil phase at high temperature.^54^ The furnace’s working temperature of 900 °C is well above the melting point of Zn and very close to the 907 °C boiling point of Zn at atmospheric pressure.^55^ Any local overheating could have evaporated Zn.

Interestingly, some metals with relatively high concentrations in the pXRF measurements of biochars, notably Mn and Fe, are not above the limits of detection in the ICP-OES measurement (Table S8). The metals may have complexed in the microwave digestion solution and been removed in the filtering step, or pXRF may overestimate the concentration of metals in the sample, again demonstrating the need to correct pXRF measurements of metals in plant matrices.

The pH, acid neutralizing capacity, cation exchange capacity, and surface area of the *O. chalcidica* biochars are shown in Table 1. The biochars are both alkaline, as is typical for plant-based biochars.^1,56^ MN900, synthesized from the MN plant master mix, exhibited higher acid neutralizing capacity and similar pH and cation exchange capacity to C900, synthesized from the C plant master mix. Both showed high pH and acid neutralizing capacity relative to other biochars in the literature; the accumulated Ca as CaCO_3_ in the leaf trichomes and K throughout the plants likely contributed to these characteristics.^40,56^ High pH and acid neutralizing capacity can enhance Ni(II) sorption from solutions with extremely acidic initial pH, since Ni(II) sorption primarily occurs above pH 5.^57^ C900 had a slightly higher surface area than MN900, though both were similar and of the magnitude expected from previous results.^17^ The N_2_ adsorption/desorption isotherms for both biochars are IUPAC Type IV isotherms with Type H4 hysteresis loops, which are often characteristic of mesoporous structures with narrow slit-like pores or some microporosity (Figure S3).^58^

**Table 1:** Characteristics of *O. chalcidica* Biochars^a^

biochar	pH	ANC_pH7_ (mmol_c_ kg^–1^)	CEC-BC (mmol_c_ kg^–1^)	CEC-NH_4_^+^ (mmol_c_ kg^–1^)	surface area (m^2^ g^–1^)
MN900	11.7	3480	314	247	175 ± 3
C900	11.5	2880	282	234	189 ± 3

a ANC_pH7_, acid neutralizing capacity; CEC-BC, cation exchange capacity by sum of exchangeable base cations at pH 7; CEC-NH_4_^+^, cation exchange capacity by displaced NH_4_^+^.

The SEM images show that both biochars have a similar structural composition: irregular monoliths with some smaller, unstructured loose material (Figures S4 and S5). The biochar pores are extended cylindrical structures with additional small, lateral openings connecting the cylinders forming a 3D honeycomb-like structure, likely resulting from the capillary systems of the plants. An open pore structure is often favorable for metal removal from a solution, as it increases the biochar–surface interface and minimizes rate limitation due to mass transfer.^59^

### Biochar Sorption Isotherms

**Figure 1:**
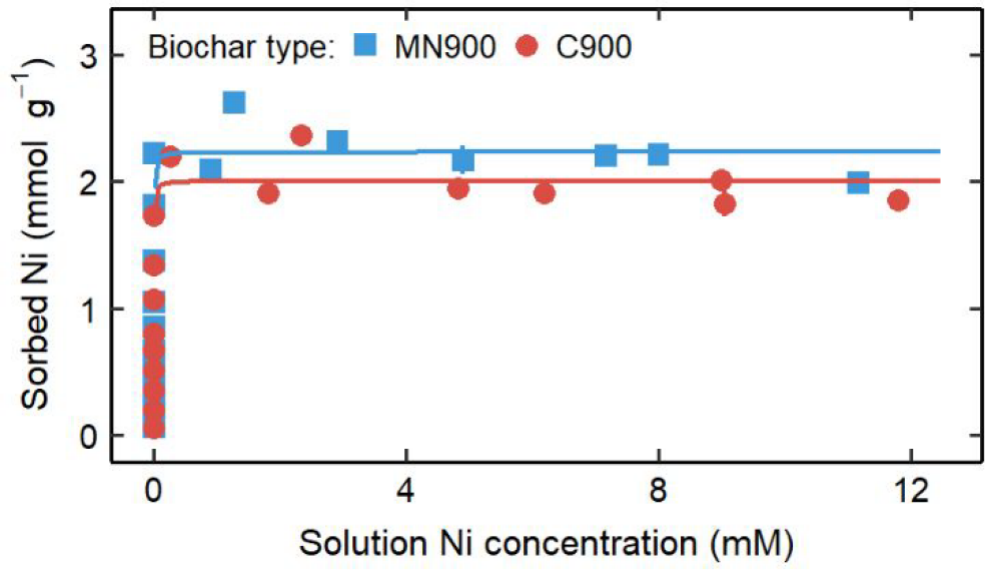
Sorption isotherms for biochars removing Ni(II) from solution. The best-fitting model lines as determined by minimum residual standard error are displayed on the graph with a solid line indicating a Langmuir isotherm. When error bars representing ±1 standard deviation are not visible, error is within the marker.

Batch sorption experiments were conducted on each biochar with solutions of nominal concentrations ranging from 0 to 20 mM Ni(II). The filtrate pH values ranged between 7.50 and 11.59 and tended to decrease with higher initial Ni(II) concentration. The amount of Ni(II) removed by each biochar was plotted against the equilibrium Ni(II) concentration to display the sorption isotherm (Figure 1). The maximum sorbed Ni(II) values and the average equilibrium pH of the corresponding solutions are given in Table 2. The isotherm equations and model fitting parameters are given in Table S9. The biochar sorption isotherms were best fit by the Langmuir model as determined by minimum residual standard error. Nonlinear analysis, as used here, was preferable to linearized analysis for the chosen models because the linearized forms could not account for complete removal of Ni from solution.^43^ Prevalent mechanisms for removal of metals from solution by biochars pyrolyzed at high temperature include precipitation, cation exchange, electrostatic interactions between cations and π bonds in the biochar, and physical adsorption.^6^ Adsorption, as described by the Langmuir model, assumes monolayer coverage at adsorption sites. Neither cation exchange nor precipitation mechanisms are consistent with this assumption. The departure of the data from the Langmuir models in Figure 1 indicates that adsorption is likely not the primary mechanism of Ni(II) removal from the system.

**Table 2:** Maximum Observed Ni(II) Sorption Values (*q*_max_) with Error Reported as ±1 Standard Deviation and Average Corresponding Equilibrium pH

biochar	*q*_max_ (mg Ni(II) g^–1^)	pH
MN900	154 ± 3	8.0
C900	139 ± 1	7.9

In comparison to the sorption maxima of other promising Ni(II) sorbents in the literature, *O. chalcidica* biochar is an excellent sorbent, likely due to its high acid neutralizing and cation exchange capacities. Of 99 natural materials compared in a recent Ni sorption survey, with sorption maxima ranging between 1 and 780 mg Ni(II) g^–1^, *O. chalcidica* biochar was outperformed by only nine materials.^57^ All of those materials were synthesized and modified in multistep processes, composited with other materials, or synthesized from a specialty feedstock that would be difficult to acquire at industrial scales (i.e., chicken eggshell, tree bark, bacteria extracts, etc.). *O. chalcidica* biochar was the highest-performing unmodified biochar with a plant feedstock. Additionally, the biochar outperformed 11 carbon nanotube materials specifically designed for divalent metal ion sorption.^60−62^ Biochar made from *O. chalcidica*, which was grown on Ni-spiked potting mix and did hyperaccumulate Ni, also demonstrated high Ni(II) sorption capacity, indicating that Ni hyperaccumulation in the feedstock does not prevent Ni(II) sorption.^17^*O. chalcidica* biochar has extremely high Ni(II) sorption capacity, the feedstock can be grown on marginal soils, and the synthesis is completed in one step; combined, these make it an excellent candidate for Ni(II) sorption from wastewater.

**Figure 2:**
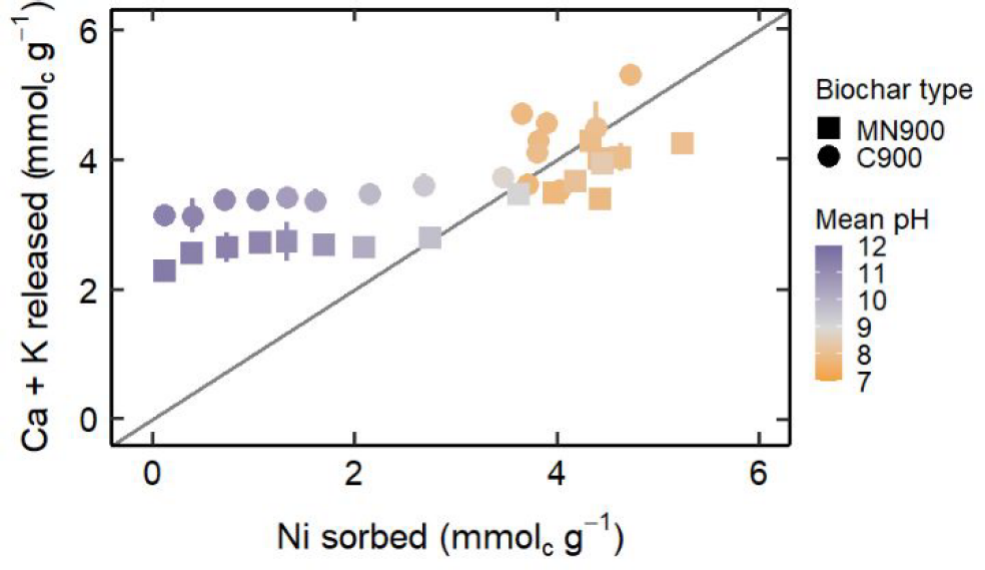
Sum of Ca^2+^ and K^+^ released by biochar into solution shown against Ni(II) removed from solution by biochar. The points are colored by mean final solution pH. The gray line is a 1:1 association line. When error bars representing ±1 standard deviation are not visible, error is within the marker.

ICP-OES analysis revealed that while Ni(II) is removed, Ca^2+^ and K^+^ accumulated by the plants during growth are released by the biochars into solution. This indicates that cation exchange is a contributory mechanism to Ni(II) removal from solution. To examine this, Ca^2+^ charge released, K^+^ charge released, and the sum of Ca^2+^ and K^+^ charge released are plotted against Ni(II) (assumed 2+) charge sorbed by the biochars and colored by final solution pH. Figure S6 shows that sorption appears independent of Ca^2+^ charge released until Ni(II) charge sorbed is approximately 3 mmol_c_ g^–1^, where Ca^2+^ charge released increases with Ni(II) charge sorbed. The increase of Ca^2+^ in solution is likely due to a combination of cation exchange and dissolution of CaCO_3_ from the biochar. Figure S7 shows that MN900 and C900 both release ∼3 mmol_c_ g^–1^ of K^+^ into solution, independent of Ni(II) sorbed. Figure 2 shows that biochar charge released was initially independent of Ni(II) charge sorbed, but that above the ∼3 mmol_c_ g^–1^ concentration, the charge sorbed and sum of charge released cluster around the 1:1 line. Taken together, this provides evidence for the following mechanism for removal of Ni(II) by *O. chalcidica* biochar from an aqueous system: Upon solution contact, all soluble salts leach and solution pH rises rapidly due to the acid neutralizing and buffering capabilities of the biochar. Removal of Ni(II) from the aqueous phase proceeds by precipitation and/or cation exchange. In solutions with pH > ∼8, precipitation may be the dominant mechanism. In solutions with pH < ∼8, Ni(II) cations are exchanged with the soluble cation fraction, which is primarily K^+^. If the Ni(II) concentration is in excess of the soluble cation concentration in the biochar, Ni(II) sorption also occurs by cation exchange of other cations from the biochar, primarily Ca^2+^, with Ni(II) from the solution. Other cations including Mg^2+^ are exchanged with Ni(II) at lower concentrations. This continues until the biochar’s sorption maximum is reached. It is likely that other sorption mechanisms including electrostatic attraction and adsorption contribute to Ni(II) removal from the system, though precipitation and cation exchange seem to be dominant.

### Biochar Sorption Kinetics

**Figure 3:**
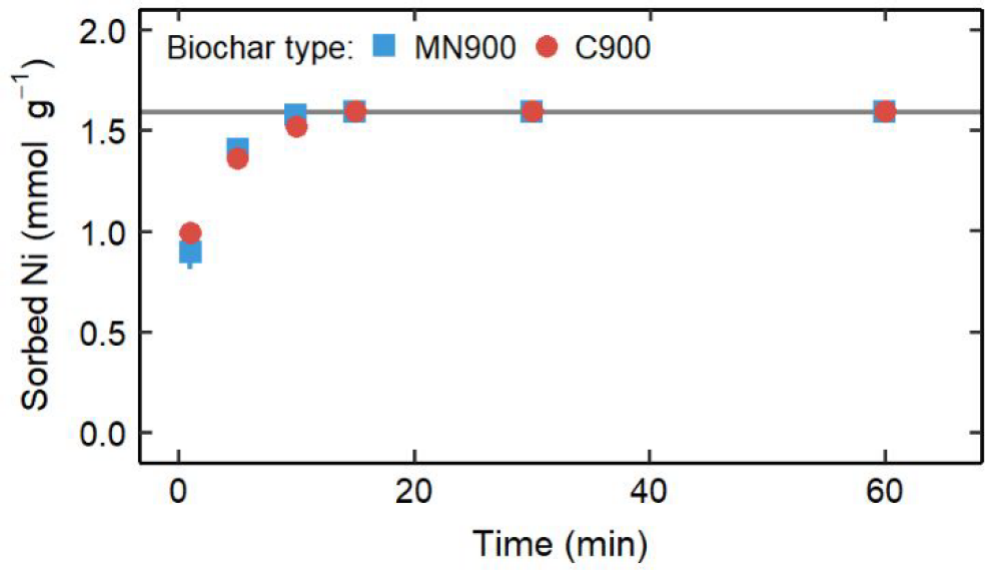
Ni(II) sorption kinetics for the first 60 min of the experiments with 5 mM Ni(II) and pH 5 in the initial solution. The gray line is the sorbed Ni(II) concentration after 24 h for both MN900 and C900. When error bars representing ±1 standard deviation are not visible, error is within the marker.

The biochar sorption kinetics were examined using a solution with 5 mM Ni(II) at initial pH 5 over 24 h. As with the isotherm experiments, Ni(II), Ca^2+^, and K^+^ showed changes in concentration throughout the experiment. Ni(II) sorption increased asymptotically over the initial 15 min of the experiment (Figure 3) to the final concentration and remained there for the remainder of the experiment (Figure S8). The Ni(II) removal efficiency was 100% for MN900 and C900; no detectable Ni(II) remained in solution after 15 min. Solution pH values were >8 by 1 min, indicating that precipitation likely contributed to Ni removal.

K^+^ was released from the biochars, with the majority released in the first minute due to its high solubility in aqueous systems (Figure S9). The K^+^ release concentration was approximately twice the initial sorbed Ni(II) concentration, consistent with a cation exchange mechanism. Ca^2+^ was steadily released for the initial 15 min of the reaction (Figure S10). This paralleled the decrease in Ni(II) concentration over the same time, indicating that it was likely involved in cation exchange in Ni(II). Low concentrations of Mg^2+^ were detected in the solution after 30 min; Mg^2+^ may have been released from a less-soluble salt or exchanged with K^+^ and Ca^2+^ over the course of the experiment. No additional metals were detected. Even though *O. chalcidica* biochar required at least 24 h to equilibrate with the solution, the Ni(II) removal was completed within 15 min, indicating that equilibrium may not be a prerequisite for maximum Ni(II) removal and that only a relatively short contact time may be required for substantial Ni(II) removal from Ni(II)-containing solutions.

### Biochar Sorption pH Dependence

**Figure 4:**
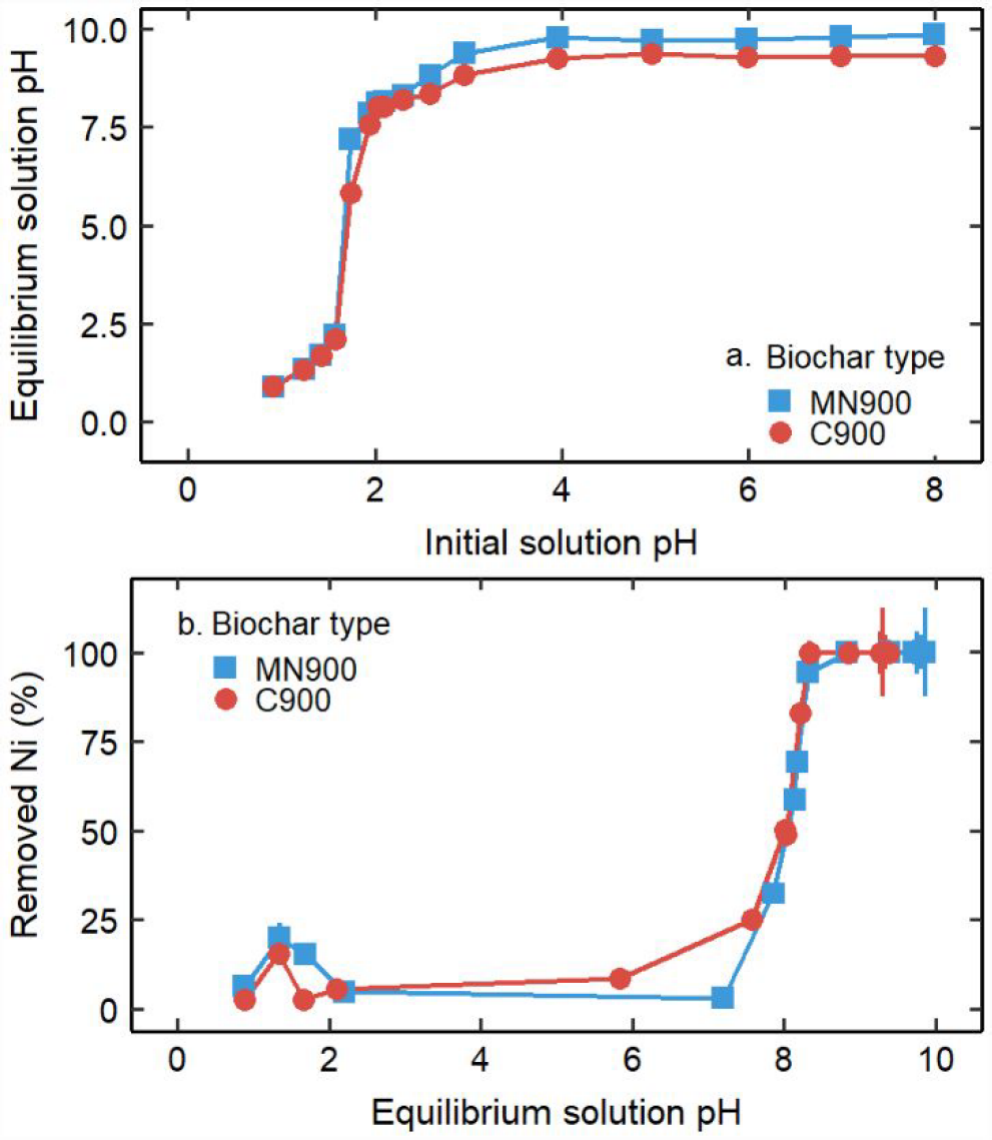
(a) Initial and equilibrium solution pH and (b) equilibrium solution pH and Ni(II) removal for an aqueous system with 5 mM Ni(II) in the initial solution. Color and shape indicate biochar type. The lower graph clearly shows the “sorption edge” for Ni(II) around pH 8 for both biochars.

The dependence of Ni(II) sorption on pH was examined by varying the starting pH of solutions containing 5 mM Ni(II). The results are plotted showing initial solution pH, final solution pH, and Ni(II) removal in percent (Figure 4). When the initial solution had a pH of 9 or greater, Ni(II) removal was 100% in all samples including the blanks, and so the data is not included in the figure. Thermodynamic considerations indicate that precipitation may be a major Ni(II) removal mechanism when the final pH > 8.^24^ When the initial pH was below 2, the final pH was less than 2.5 and the Ni(II) removal was low. This was as expected; 10 mL of solution with pH below 2 corresponds to a hydronium ion amount greater than the acid neutralizing capacity of 0.05 g of biochar. Ni(II) sorption to other biochar materials without precipitation has been reported in the literature to reach a maximum between pH 5 and 7 due to decreased competition with hydronium ions for binding sites and electrostatic repulsion.^57^ Both the equilibrium solution pH and Ni(II) removal increased rapidly between initial pH 2 and pH 2.5, and the Ni sorption edge is apparent around an equilibrium pH of 7.5 where Ni(OH)_2_ precipitation was predicted to begin (Figure S11). The MN900 biochar buffered solutions to approximately pH 9.8, and the C900 biochar buffered solutions to approximately pH 9.3. Both removed 100% of Ni(II). While Ni(II) was successfully removed from most experimental solutions, these results indicate that the tested ratio of biochar to highly acidic (<pH 2), high ionic strength aqueous solutions may be insufficient to remove Ni(II) from the system. Instead, the ratio of biochar to solution may have to be increased and optimized for application in specific environmental or industrial systems.

### Biochar Sorption from Complex Mixtures

After examining removal of Ni(II) from solution by *O. chalcidica* biochar, we examined removal of metals from simulated *O. chalcidica* leachate and simulated Ni electroplating rinsewater.

The simulated *O. chalcidica* leachate contained metals and malic, malonic, citric, acetic, and oxalic acids.^47^ Of the metals, only Fe was not detected in the ICP-OES analysis. The biochars did not remove Ni(II) from the solution (Figure S12a). This was unexpected; the average final pH of the MN900 and C900 solutions were 9.11 and 9.04, respectively. According to speciation calculations of Ni(II) under similar conditions in the literature, precipitation of Ni(OH)_2_ from the system was expected to begin at pH 8.5 and be approximately 50% complete by pH 9.^47^ However, Ni(II) removal by precipitation did not occur despite elevated pH, indicating that Ni(II) was likely chelated by carboxylic acids in the simulated *O. chalcidica* leachate solution. Previous studies have also observed this lack of Ni(OH)_2_ precipitation at elevated pH in *O. chalcidica* leachate and attributed it to the presence of organic matter or chelators from the plant material in the system. However, organic matter or chelates from the plant material are unlikely to survive pyrolysis at 900 °C and so are unlikely to be present in the experimental system. Instead, our results demonstrate that involvement of low molecular weight carboxylic acids in the system prevents selective precipitation of Ni(II) at the examined time scale.^47,63^

Due to the lack of Ni(II) precipitation, we tested the sorption of a metals-only simulated *O. chalcidica* leachate on the *O. chalcidica* biochars (Figure S12b). MN900 and C900 removed 95% and 88% of Ni(II) in the system and had final average pH values of 7.98 and 7.97, respectively. This demonstrates that *O. chalcidica* biochar can remove Ni(II) from simulated *O. chalcidica* leachate when Ni(II) is a free cation in solution, but not when it is chelated in a strongly bound organic acid-metal complex. The specific sorption and pH values are comparable to the final values of high Ni trials in the Ni sorption isotherm experiment, demonstrating that *O. chalcidica* biochar can remove comparable levels of Ni^2+^ in simple and more complex aqueous systems without high concentrations of soluble chelators.

**Figure 5:**
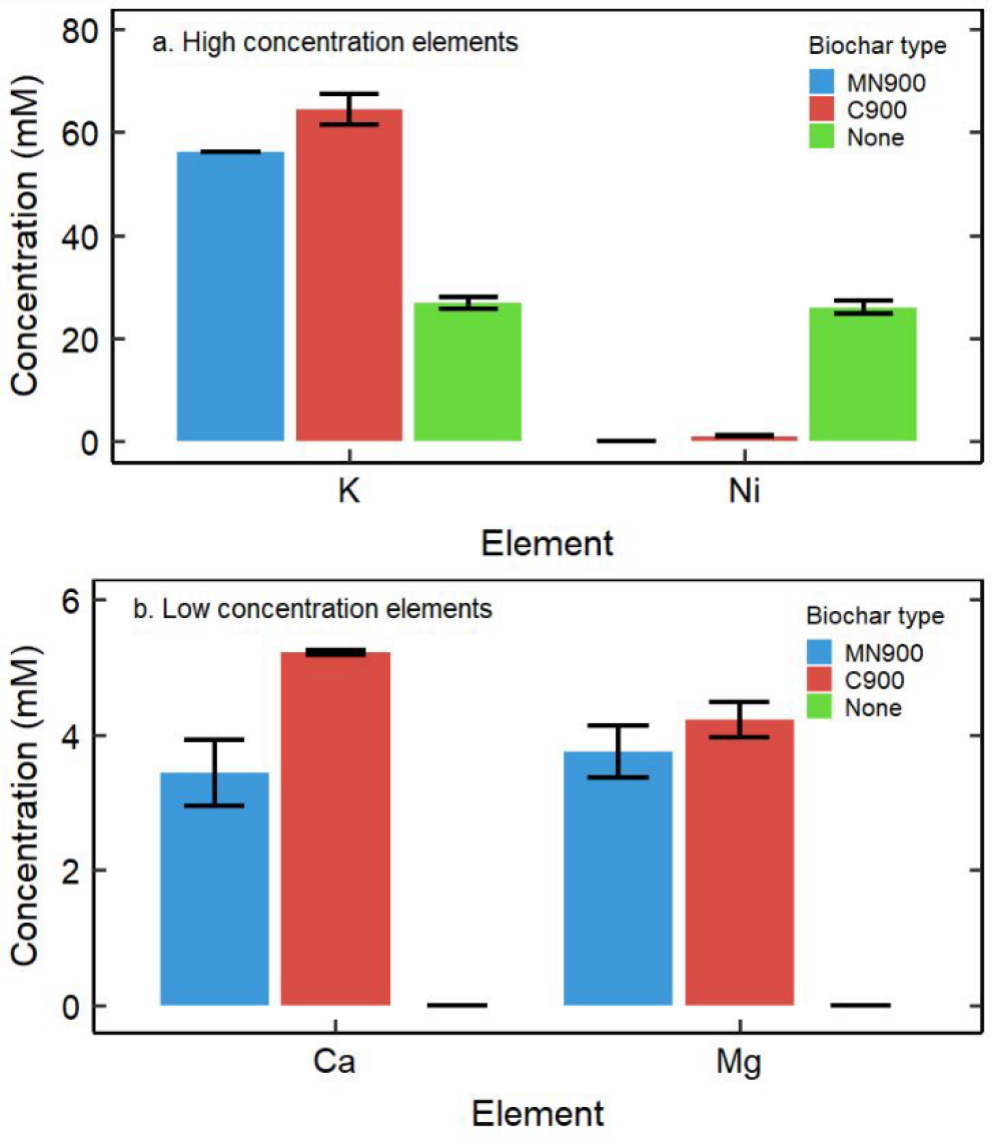
Elemental composition of aqueous phase after 24 h of contact between simulated Ni electroplating rinsewater and the indicated biochar, separated by (a) high concentration elements and (b) low concentration elements.^48^ “None” refers to experimental controls with no biochar added. Columns represented by black lines indicate that the element concentration was below the limit of detection. Error bars represent ±1 standard deviation.

The capacity of *O. chalcidica* biochar to remove metals from a simulated Ni electroplating rinsewater solution was tested (Figure 5). The final pH values were 8.64 and 8.37 for MN900 and C900, respectively. As expected, the biochars released K^+^, Ca^2+^, and Mg(II); higher concentrations of cations released compared to previous experiments presented in this work are due to the higher amount of biochar used in the experiment. No Ni was detected in the final solution with the MN900 biochar, while 1 mM Ni remained in the final solution with the C900 biochar. This correlates with the calculated Ni speciation diagram, since at pH 8.37 Ni(II) is predicted to be transitioning between the solids Ni_4_(OH)_6_SO_4_ and Ni(OH)_2_, and that transition is complete by pH 8.64 (Figure S13). This also suggests that precipitation was a dominant mechanism of Ni(II) removal from this system. Additional testing should be completed with actual Ni electroplating rinsewater, as it can contain manufacturer-specific additives to make the products more visually pleasing.^48^ However, this work demonstrates that removing Ni(II) from Ni-containing industrial wastewater is a viable method to produce Ni-enhanced bio-ore and that the wastewater stream can be simultaneously neutralized and detoxified through the addition of *O. chalcidica* biochar.

### Biochar Postsorption Characterization

Biochar samples were acid digested after the sorption isotherm experiments with 10 mM Ni solution (Table S8). As expected, results show decreased Ca, K, and Mg and increased Ni concentrations in the biochar. Ni concentrations in the spent biochar digest were lower than the calculated values of Ni removal from solution. This could indicate that Ni as Ni(OH)_2_ precipitated or adsorbed on the glass vial walls due to the solution’s alkalinity during the sorption experiment and was thus not recoverable from the biochar, that Ni could not be fully removed from the biochar during acid digestion, or that Ni was removed from solution in the acid digestion process.

XRD results revealed the existence of crystalline calcite (PDF #99-000-0548), hydroxylapatite or chlorapatite (PDF #99-000-1643), and Ni (PDF #01-077-3085) (Figure S14). Crystalline Ni(OH)_2_ or NiO was not observed, possibly due to background at low angles or a lack of clear crystalline structure upon precipitation or cation exchange; amorphous phases are not identifiable by XRD.^64,65^

SEM-EDS results did not detect Ni in biochar samples not subjected to Ni sorption experiments. They confirm XRD results; crystalline Ca structures corresponding to the calcite phase (Figure S15) and Ca–P structures corresponding to the apatite phase (Figure S16) were present. No Ni-associated crystalline structures were discovered, though Ni was visibly distributed across the samples (Figures S16 and S17). Additional SEM-EDS results are given in the data deposit associated with this work.

## Conclusion

This study demonstrates the sorption capabilities of biochar synthesized at 900 °C from *O. chalcidica* biomass grown in sandy Minnesota soils from a mining district. *O. chalcidica* grew on nonserpentine soils but did not hyperaccumulate Ni due to a lack of Ni in the soil. However, the *O. chalcidica* plants accumulated high concentrations of Ca and K, which enhanced acid neutralizing and metal-removing capacities of the resultant biochars.

The specific sorption capacity of *O. chalcidica* biochar for Ni(II) is, to the best of our knowledge, within the top 10% of any sorbent and the highest of any unmodified, noncomposite biochar with a plant material feedstock.^57^ The biochar performed best with an initial solution above pH 2 and a final solution pH at or below pH 8, as expected for removal of Ni(II) from solution, where precipitation of Ni(OH)_2_ could begin but cation exchange could occur as well. Ni(II) sorption was quite fast, occurring within the first 15 min of the experiment. However, Ni(II) sorption was only possible when Ni was not chelated with organic acids, which may limit Ni(II) sorption from wastewaters containing high concentrations of organo-metal complexes. Biochar from *O. chalcidica* pyrolyzed at 900 °C also removed Ni(II) from simulated Watts bath Ni electroplating rinsewater, demonstrating the feasibility of creating an enhanced Ni bio-ore from an industrial waste product.

## Data Availability

The underlying data for this work has been deposited in the Iowa Research Online (IRO) institutional data repository at https://doi.org/10.25820/data.006176 for future reuse under an Open Data Commons Attribution License (ODC-By). There are no registration or fee requirements to download the underlying data set for this work.

## Supporting Information Available

Additional numeric results, graphs, SEM images, and SEM-EDS images (PDF)
