# Supplementary material for: Nickel Hyperaccumulator Biochar Sorbs Ni(II) from Water and Wastewater to Create an Enhanced Bio-ore
Source: ACS Environ Au. 2022 Sep 16;3(1):24–33. doi: 10.1021/acsenvironau.2c00028 (PMC9853938; doi:10.1021/acsenvironau.2c00028)
Supplement: Supplementary file 1 — vg2c00028_si_001.pdf [file vg2c00028_si_001.pdf]

Supporting Information

# Nickel hyperaccumulator biochar sorbs Ni(II) from water and wastewater to create an enhanced bio-ore

Rachel A. Smoak*†‡, Jerald L. Schnoor†‡

†Department of Civil and Environmental Engineering, University of Iowa, 4105 Seamans Center for the Engineering Arts and Sciences, Iowa City, Iowa 52242, USA

‡IIHR – Hydroscience and Engineering, University of Iowa, 100 C. Maxwell Stanley Hydraulics Laboratory, Iowa City, Iowa, 52242, USA

**Table S1.** Components of Miracle-Gro Water Soluble All Purpose Plant Food as described on the product label.

| Nutrient | Mass fraction (mg $g^{-1}$) | Parent compound(s) |
|---|---|---|
| Ammoniacal nitrogen | 35 | Ammonium sulfate |
| Urea nitrogen | 205 | Urea, urea phosphate |
| Available phosphate ($P_2O_5$) | 80 | Potassium phosphate, urea phosphate |
| Soluble potash ($K_2O$) | 160 | Potassium chloride |
| Boron (B) | 0.2 | Boric acid |
| Water soluble copper (Cu) | 0.7 | Copper sulfate |
| Chelated iron (Fe) | 1.5 | Iron EDTA |
| Chelated manganese (Mn) | 0.5 | Manganese EDTA |
| Molybdenum (Mo) | 0.005 | Sodium molybdate |
| Water soluble zinc (Zn) | 0.6 | Zinc sulfate |

**Table S2.** Concentrations of chemical components in simulated *O. chalcidica* plant leachate in DI water based on Guilpain *et al.* (2018) and simulated nickel electroplating rinse solution based on Benvenuti *et al.* (2014) used in complex solution sorption experiments. Cells containing "." indicate that the compound was not required for the given solution.

| Compound | Simulated leachate (mM) | Simulated Ni electroplating (mM) |
|---|---|---|
| $KNO_3$ | 25 | . |
| $Ni(NO_3)_2 \cdot 6H_2O$ | 10 | . |
| $MgSO_4 \cdot 7H_2O$ | 8 | . |
| $Ca(NO_3)_2 \cdot 6H_2O$ | 7 | . |
| $FeCl_3 \cdot 6H_2O$ | 0.02 | . |
| $C_4H_6O_5$ (malic acid) | 19 | . |
| $C_3H_4O_4$ (malonic acid) | 12 | . |
| $C_6H_8O_7$ (citric acid) | 4 | . |
| $C_2H_4O_2$ (acetic acid) | 3 | . |
| $C_2H_2O_4$ (oxalic acid) | 1 | . |
| $NiCl_2 \cdot 6H_2O$ | . | 5 |
| $NiSO_4 \cdot 6H_2O$ | . | 18 |
| $H_3BO_3$ | . | 7 |

**Table S3.** Pre- and post-experiment soil pH and metal content measured by pXRF for soils gathered from Minnesota (MN) and potting mix (PM) master mixes. pXRF limits of detection (LODs) determined by the manufacturer are also shown.

| Condition | Soil | pH | Concentration (mg kg$^{-1}$ dry weight) | | | | | | | | |
|---|---|---|---|---|---|---|---|---|---|---|---|
| | | | Ni | Sr | Cu | Zn | Mn | Fe (g kg$^{-1}$) | Ti (g kg$^{-1}$) | Cr | Zr |
| Pre-experiment | MN | 6.1 | 70 ± 10 | 530 ± 40 | 26 ± 7 | 32 ± 4 | 570 ± 70 | 39 ± 5 | 4.4 ± 0.5 | 140 ± 30 | 50 ± 10 |
| | PM | 5.3 | | 56 ± 5 | | 28 ± 2 | 160 ± 20 | 2.7 ± 0.4 | | | 13 ± 2 |
| Post-experiment | MN | 7.8 | 70 ± 20 | 560 ± 20 | 250 ± 20 | 120 ± 20 | 600 ± 100 | 38 ± 7 | 4 ± 2 | 200 ± 100 | 60± 10 |
| | PM | 7.5 | | 660 ± 30 | 630 ± 60 | 210 ± 40 | 90 ± 20 | 3 ± 1 | | | 9 ± 6 |
| pXRF LOD | | | 10 | 5 | 10 | 5 | 10 | 0.01 | 0.01 | 10 | 5 |

Cells left blank indicate that the element was not detected

**Table S4.** Results from the Minnesota Valley Testing Laboratories, Inc. soil analysis on the MN soil

| Soil Sample | pH | Salinity (mmhos/cm) | Buffer Index | Organic Matter (mg g$^{-1}$) | Sand (mg g$^{-1}$) | Silt (mg g$^{-1}$) | Clay (mg g$^{-1}$) | Cation exchange capacity (mmol$_c$/100 g) |
|---|---|---|---|---|---|---|---|---|
| A | 6.2 | 1.1 | 7.2 | 6 | 925 | 50 | 25 | 8.8 |
| B | 5.9 | 0.5 | 7.2 | 6 | 925 | 50 | 25 | 8.9 |
| C | 6.3 | 0.3 | 7.2 | 8 | 900 | 75 | 25 | 7.5 |
| D | 6.0 | 0.2 | 7.2 | 8 | 900 | 75 | 25 | 6.4 |
| E | 6.1 | 0.3 | 7.2 | 8 | 875 | 100 | 25 | 6.4 |
| Average | 6.1 | 0.5 | 7.2 | 7 | 905 | 70 | 25 | 7.6 |

| Soil Sample | Base saturation (mg g$^{-1}$) | | | | | $NO_3$-N (mg kg$^{-1}$) | $SO_4$-S (mg kg$^{-1}$) | P (mg kg$^{-1}$) |
|---|---|---|---|---|---|---|---|---|
| | Ca | Mg | K | Na | H | | | |
| A | 496 | 243 | 19 | 241 | 0.0 | 32.9 | 371 | 7 |
| B | 487 | 236 | 20 | 258 | 0.0 | 73.8 | 371 | 6 |
| C | 508 | 264 | 21 | 208 | 0.0 | 25.7 | 270 | 8 |
| D | 630 | 274 | 21 | 7.6 | 0.0 | 4.3 | 48 | 10 |
| E | 609 | 266 | 21 | 104 | 0.0 | 13.0 | 94 | 11 |
| Average | 546 | 257 | 20 | 177 | 0 | 29.9 | 231 | 8 |

| Soil Sample | K (mg kg$^{-1}$) | Ca (mg kg$^{-1}$) | Na (mg kg$^{-1}$) | Mg (mg kg$^{-1}$) | Fe (mg kg$^{-1}$) | Cu (mg kg$^{-1}$) | Mn (mg kg$^{-1}$) | B (mg kg$^{-1}$) | Zn (mg kg$^{-1}$) |
|---|---|---|---|---|---|---|---|---|---|
| A | 66 | 872 | 488 | 261 | 14.1 | 1.7 | 1.2 | 0.8 | 0.6 |
| B | 68 | 864 | 526 | 256 | 14.8 | 1.0 | 1.9 | 0.9 | 0.4 |
| C | 60 | 759 | 357 | 241 | 14.9 | 1.1 | 1.4 | 0.9 | 0.4 |
| D | 51 | 803 | 111 | 213 | 12.9 | 2.8 | 1.0 | 0.4 | 0.8 |
| E | 52 | 776 | 152 | 207 | 13.2 | 1.6 | 1.7 | 0.5 | 0.5 |
| Average | 59 | 815 | 327 | 236 | 14.0 | 1.6 | 1.4 | 0.7 | 0.5 |

Salts were measured as soluble salt concentrations. P and K were measured using the Mehlich III procedure. Ca, Na, and Mg were measured as soluble concentrations using ammonium acetate extraction. Fe, Cu, Mn, and Zn were measured as soluble concentrations with DTPA extraction. B was measured as soluble concentration with DPTA-sorbitol extraction. A full description of the procedures used can be found at http://mvtl.com/_static/web/assets/media/pdf/soil-nutrient.pdf.

**Table S5.** Concentrations of metals extractable by 0.01 M $CaCl_2$ and 1 M HCl from pre-experiment potting mix (pre-PM) post-experiment potting mix (post-PM), and post-experiment MN soil (post-MN) reported in mg metal per kg dry soil. The limit of detection (LOD) is also reported; the LOD for Ni, Ti, and Zn were 160, 63, and 52 mg $kg^{-1}$ for the $CaCl_2$ extraction and 480, 190, and 155 mg $kg^{-1}$ for the HCl extraction, respectively.

| Extraction Method | Soil | Ca (g $kg^{-1}$) | Cu (mg $kg^{-1}$) | K (g $kg^{-1}$) | Mg (g $kg^{-1}$) | Sr (mg $kg^{-1}$) | Fe (g $kg^{-1}$) |
|---|---|---|---|---|---|---|---|
| $CaCl_2$ | pre-PM | 0.11 ± 0.7 | | 1.27 ± 0.02 | 1.99 ± 0.03 | | |
| | post-PM | 0.78 ± 0.9 | | 1.27 ± 0.01 | 31.7± 0.04 | 73 ± 2 | |
| | post-MN | 0.27 ± 0.8 | | | | | |
| | LOD | 0.045 | 30 | 0.130 | 0.052 | 43 | 0.028 |
| HCl | pre-PM | 19.6 ± 0.2 | 107 ± 1 | 1.53 ± 0.02 | 4.75 ± 0.05 | | 713 ± 8 |
| | post-PM | 26.7 ± 0.4 | 256 ± 3 | 1.36 ± 0.01 | 8.53 ± 0.07 | 580 ± 5 | 907 ± 6 |
| | post-MN | 1.13 ± 0.01 | | | 1.65 ± 0.02 | | 3340 ± 40 |
| | LOD | 0.136 | 91 | 0.389 | 0.157 | 129 | 83 |

Cells left blank indicate that the element was below the limit of detection

**Table S6.** Metals concentrations reported by ICP-OES and limits of detection (LOD) after acid microwave digestion of a greenhouse water sample. The LODs of Cu, Ni, Zn, and Sr were 0.05, 0.27, 0.08, and 0.05 mM, respectively.

| Sample | Ca (mM) | K (mM) | Mg (mM) |
|---|---|---|---|
| Greenhouse water | 2.58 ± 0.03 | 0.47 ± 0.01 | 2.13 ± 0.02 |
| LOD | 0.11 | 0.33 | 0.22 |

**Table S7.** Plant master mix and biochar metal concentrations measured by pXRF for selected metals. pXRF limits of detection determined by the manufacturer are also shown.

| Sample Type | Sample | Concentration (mg kg$^{-1}$ dry weight) | | | | | | | | |
|---|---|---|---|---|---|---|---|---|---|---|
| | | **Mn** | **Sr** | **Fe** | **Ni** | **Zn** | **Cu** | **Rb** | **Co** | **Ag** |
| Plant | MN | 210 ± 30 | 280 ± 20 | 270 ± 50 | 57 ± 5 | 130 ± 20 | 32 ± 6 | 15 ± 2 | | 20 ± 5 |
| | C | 220 ± 20 | 290 ± 30 | 180 ± 30 | | 400 ± 30 | 17 ± 2 | 15 ± 2 | | 18 ± 4 |
| Biochar | MN900 | 640 ± 60 | 1180 ± 90 | 560 ± 80 | 195 ± 6 | 32 ± 1 | 114 ± 4 | 64 ± 5 | 32 ± 5 | 20 ± 10 |
| | C900 | 540 ± 60 | 1310 ± 70 | 360 ± 20 | | 57 ± 3 | 74 ± 5 | 70 ± 3 | | 20 ± 10 |
| pXRF LOD | | 10 | 5 | 10 | 10 | 5 | 10 | 5 | 10 | 10 |

Cells left blank indicate that the element was not detected

**Table S8.** Plant master mix, biochar, and post-experiment Ni10 sorbed biochar metal concentrations measured by ICP-OES after acid-assisted microwave digestion expressed per kilogram of dry material. Limits of detection (LODs) are also reported. The LODs for Fe were 105, 61, and 23 mg kg$^{-1}$ and the LODs for Mn were 164, 95, and 36 mg kg$^{-1}$ for the plant, biochar, and sorbed biochar samples, respectively.

| Sample Type | Sample | Ca (g kg$^{-1}$) | K (g kg$^{-1}$) | Mg (g kg$^{-1}$) | Ni (g kg$^{-1}$) |
|---|---|---|---|---|---|
| Plant | MN | 23 ± 1 | 18 ± 1 | | |
| | C | 22 ± 1 | 23 ±1 | | |
| | LOD | 0.2 | 0.5 | 0.2 | 0.6 |
| Biochar | MN900 | 75 ± 1 | 77 ± 1 | 8.9 ± 0.1 | |
| | C900 | 100 ± 1 | 120 ± 1 | 9.7 ± 0.1 | |
| | LOD | 0.1 | 0.3 | 0.1 | 0.3 |
| Sorbed biochar | MN900-Ni10 | 52 ± 1 | | | 74 ± 2 |
| | C900-Ni10 | 57 ± 1 | | | 64 ± 1 |
| | LOD | 0.04 | 0.11 | 0.04 | 0.1 |

Cells left blank indicate that the element concentration was below the LOD

**Table S9.** Isotherm model fitting parameters for removal of Ni(II) by each biochar.

| | | | | | | | | | | | |
|---|---|---|---|---|---|---|---|---|---|---|---|
| **Freundlich model:** $q_e = K_F C_e^{1/n}$ | | | | | | | | | | | |
| Biochar | $K_F$ | $K_F$ SE | $K_F$ p | $n$ | $n$ SE | $n$ p | RSE | DF | | | |
| MN900 | 1.90 | 0.174 | 1.48e-8 | 9.99 | 2.58 | 1.52e-3 | 0.593 | 15 | | | |
| C900 | 1.68 | 0.135 | 2.56e-9 | 9.85 | 2.35 | 7.78e-4 | 0.483 | 15 | | | |
| **Langmuir model:** $q_e = \frac{q_m b C_e}{(1+bC_e)}$ | | | | | | | | | | | |
| Biochar | $q_m$ | $q_m$ SE | $q_m$ p | $b$ | $b$ SE | $b$ p | RSE | DF | | | |
| MN900 | 2.23 | 0.213 | 2.71e-8 | 694 | 255 | 1.58e-2 | 0.563 | 15 | | | |
| C900 | 2.00 | 0.149 | 9.33e-10 | 600. | 194 | 7.30e-3 | 0.421 | 15 | | | |
| **Redlich-Peterson model:** $q_e = \frac{K_{RP} C_e}{(1+a_{RP} C_e^{\beta})}$ | | | | | | | | | | | |
| Biochar | $K_{RP}$ | $K_{RP}$ SE | $K_{RP}$ p | $a_{RP}$ | $a_{RP}$ SE | $a_{RP}$ p | $\beta$ | $\beta$ SE | $\beta$ p | RSE | DF |
| MN900 | 1280 | 576 | 4.41e-2 | 539 | 313 | 1.07e-1 | 1.05 | 0.109 | 1.57e-7 | 0.579 | 14 |
| C900 | 1010 | 317 | 6.47e-3 | 478 | 178 | 1.77e-2 | 1.05 | 0.061 | 9.17e-11 | 0.429 | 14 |

SE: standard error p: p-value RSE: residual standard error DF: model degrees of freedom

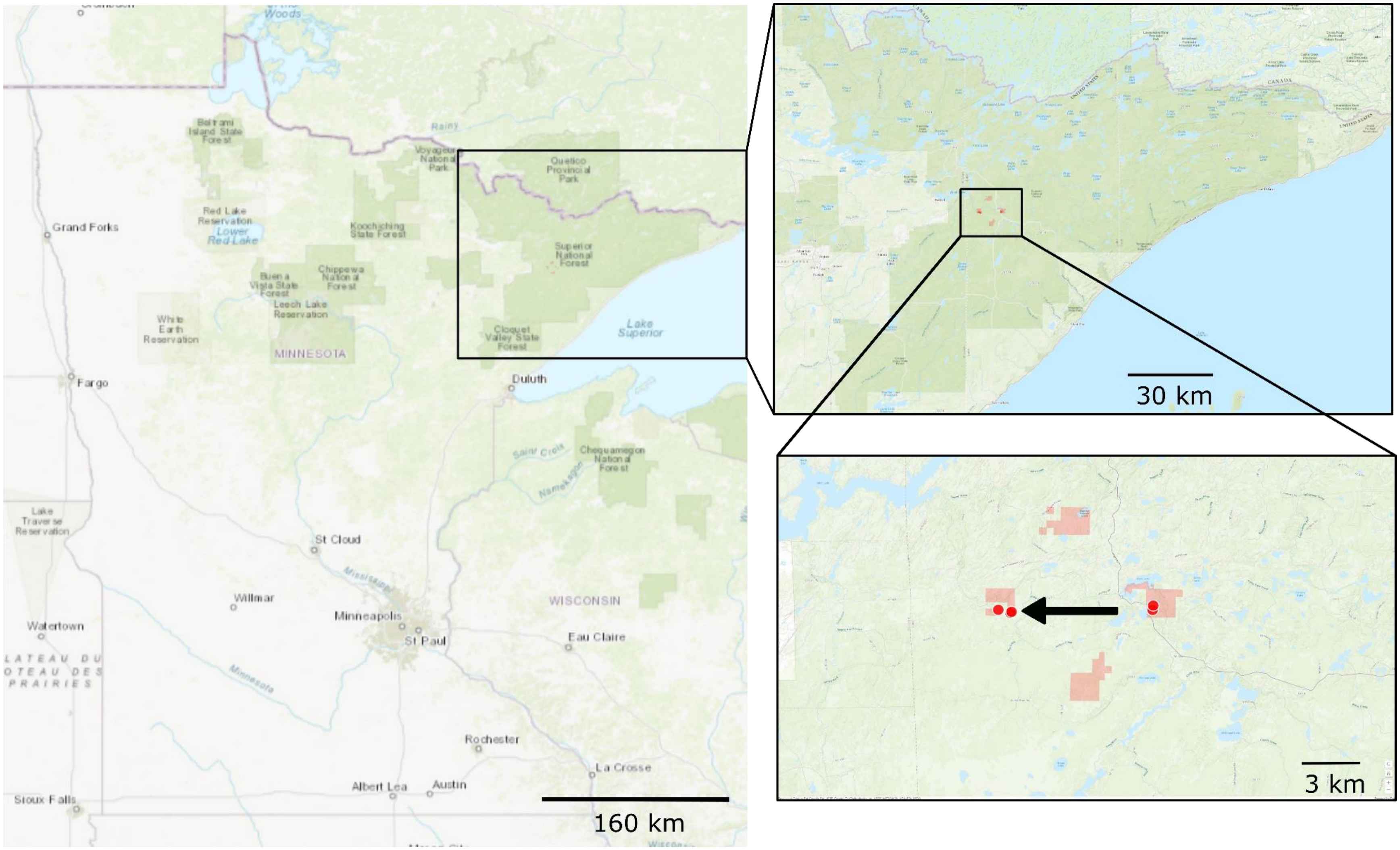


**Figure S1.** A map of soil collection areas in Minnesota, U.S.A. In the finest-scaled map, the shaded red areas are where soil surveying was permitted, the rod dots are sites that were surveyed with pXRF, and the black arrow points to the site where the final soil was collected.

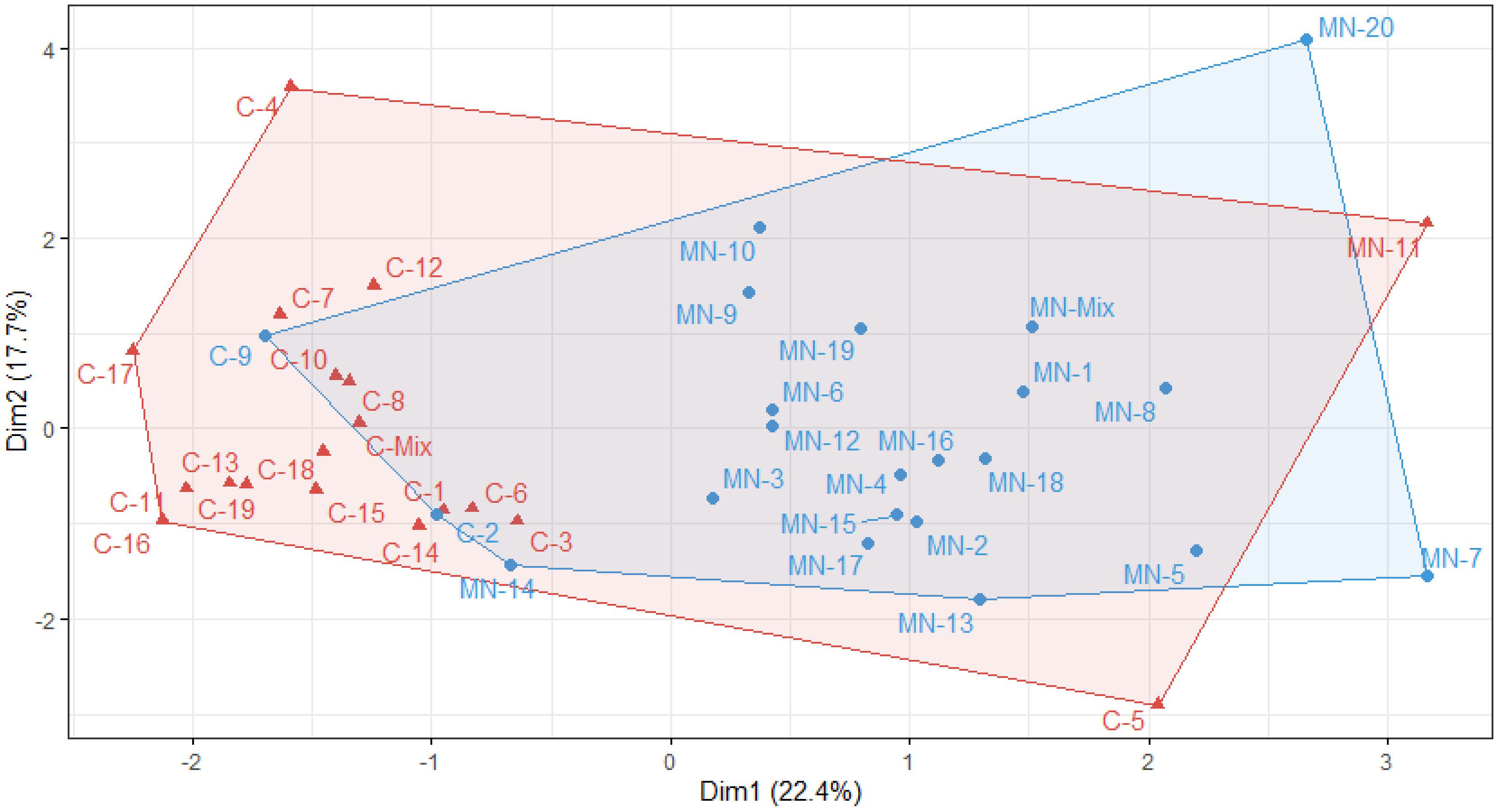


**Figure S2.** A k-means cluster plot of the pXRF individual plant metal measurements. Concentrations of elements below the limit of detection (LOD) were replaced with the LOD. Control plants 2 and 9 clustered with the Minnesota plants, and Minnesota plant 11 clustered with the control plants. Because their biomasses were so low, these plants were excluded from the MN and C plant master mixes.

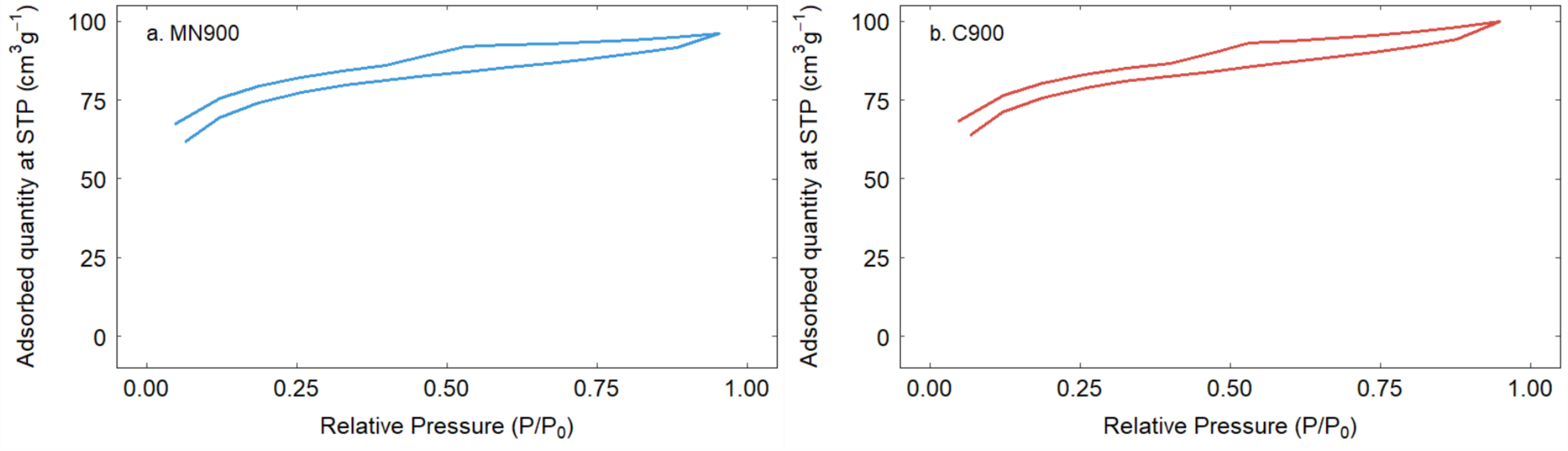


**Figure S3.** $N_2$ adsorption/desorption isotherms at 77 K for: a. MN900, b. C900. In both cases, the desorption isotherm has higher adsorbed quantities for the given relative pressures.

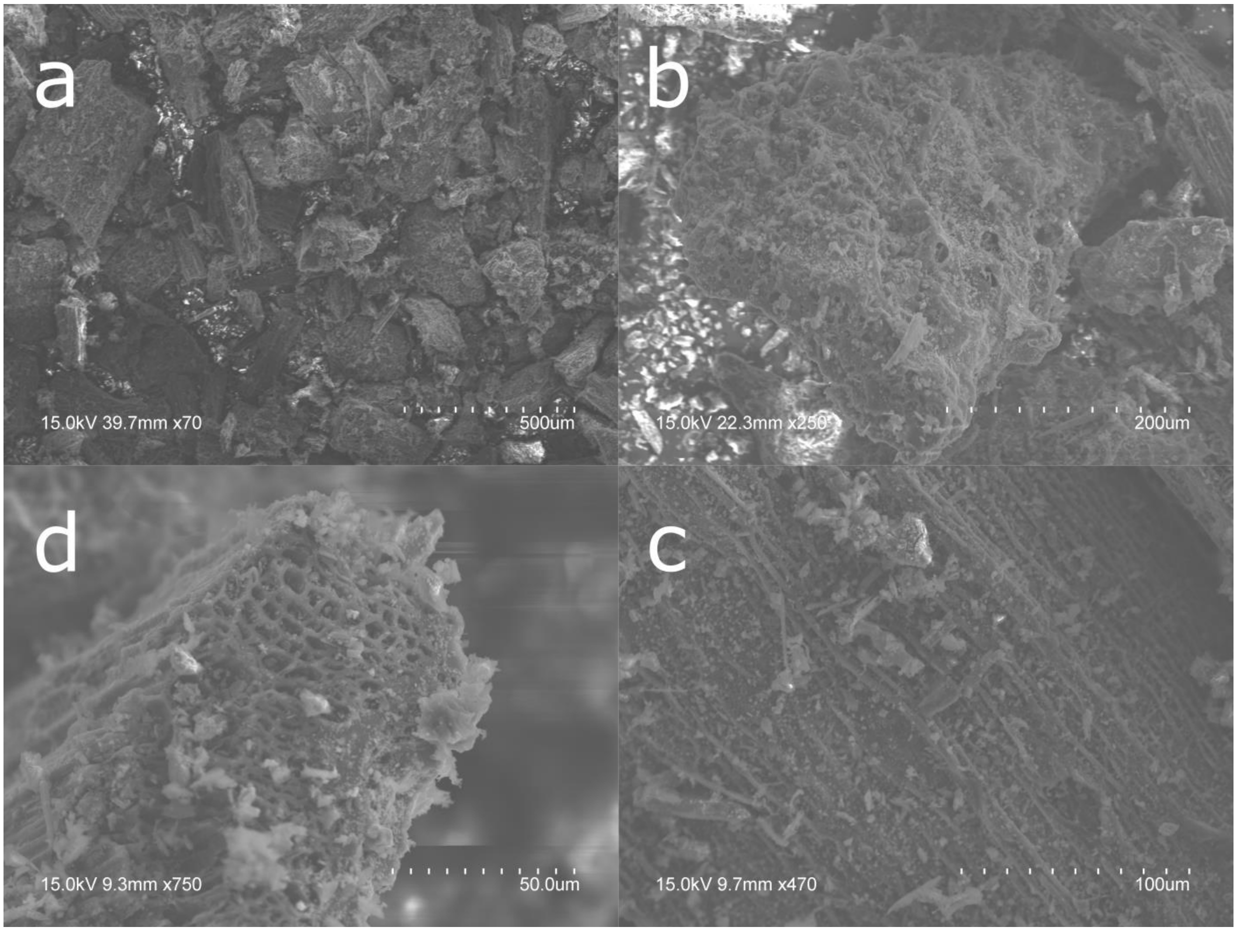


**Figure S4**. SEM images of MN900 biochar, clockwise with increasing magnification: a. gross structure; b. overall structure of one monolith; c. side surface structure; d. end surface structure with pores visible.

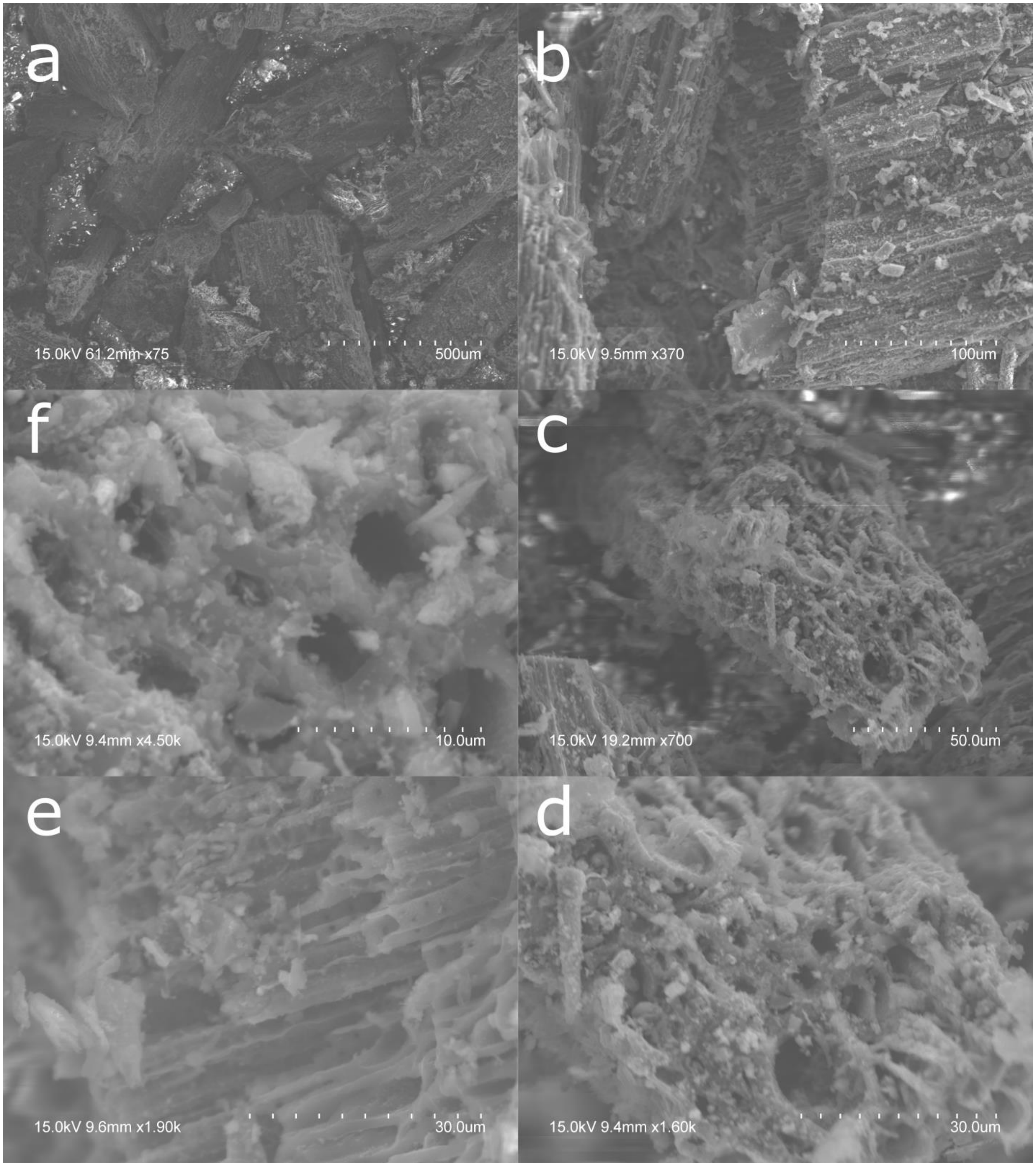


**Figure S5**. SEM images of C900 biochar, clockwise with increasing magnification: a. gross structure; b. monolith with broken edge; c. face of a monolith; d. panel c, magnified; e. panel b, magnified; f. panel d, magnified.

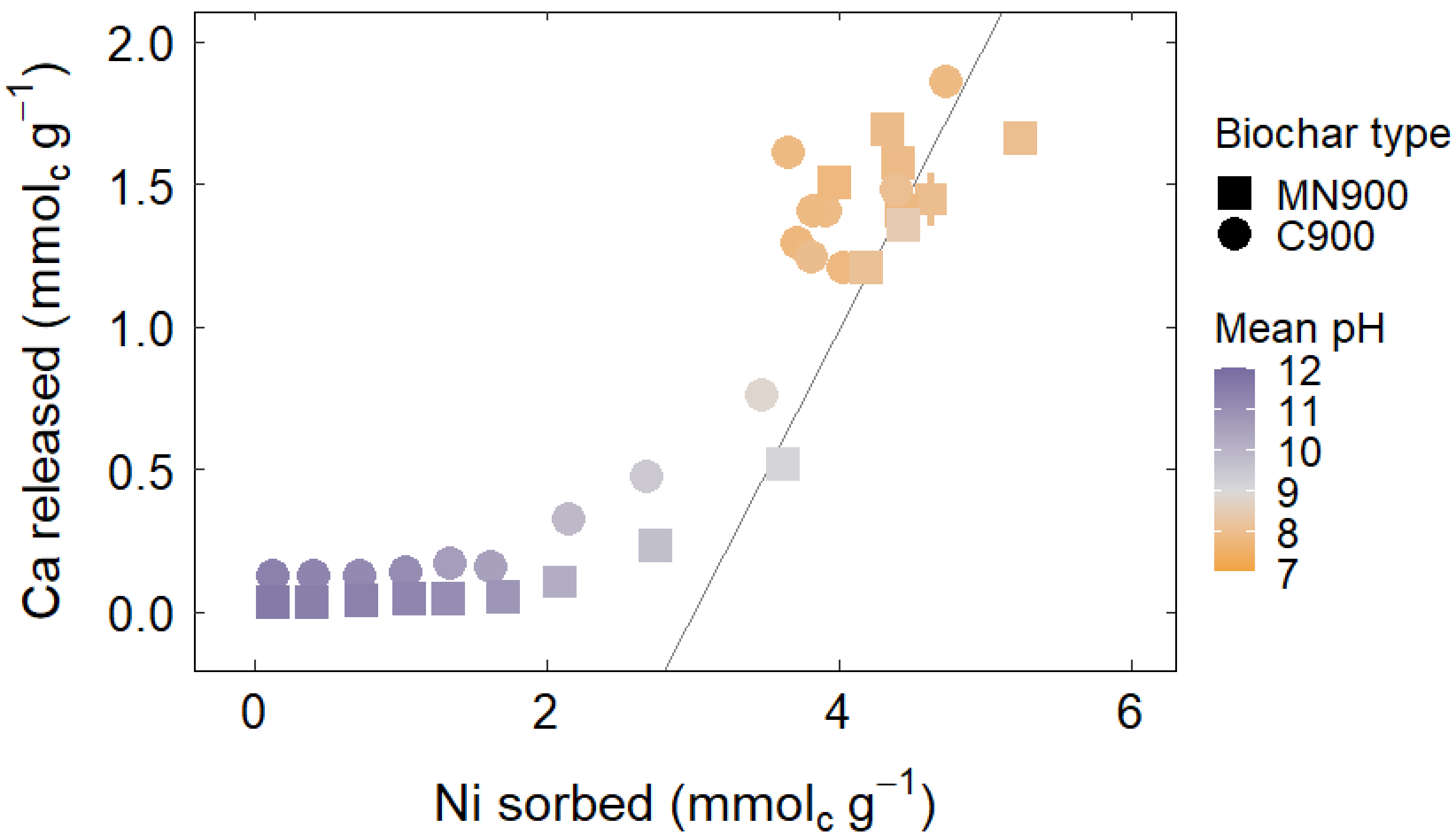


**Figure S6.** $Ca^{2+}$ released by biochar into solution shown against Ni(II) removed from solution by biochar. $Ca^{2+}$ ions are scarcely released from the biochar until Ni(II) sorption reaches 2 $mmol_c\ g^{-1}$. After 4 $mmol_c\ g^{-1}$ of Ni(II) is sorbed, ion exchange of Ni(II) for $Ca^{2+}$ appears to dominate (1:1 slope shown by gray line, intercept y = -3). The points are colored by final solution pH. pH decreases as Ni sorption increases, indicating that mechanisms other than precipitation likely contribute to Ni removal. When error bars representing ± one standard deviation are not visible, error is within the marker.

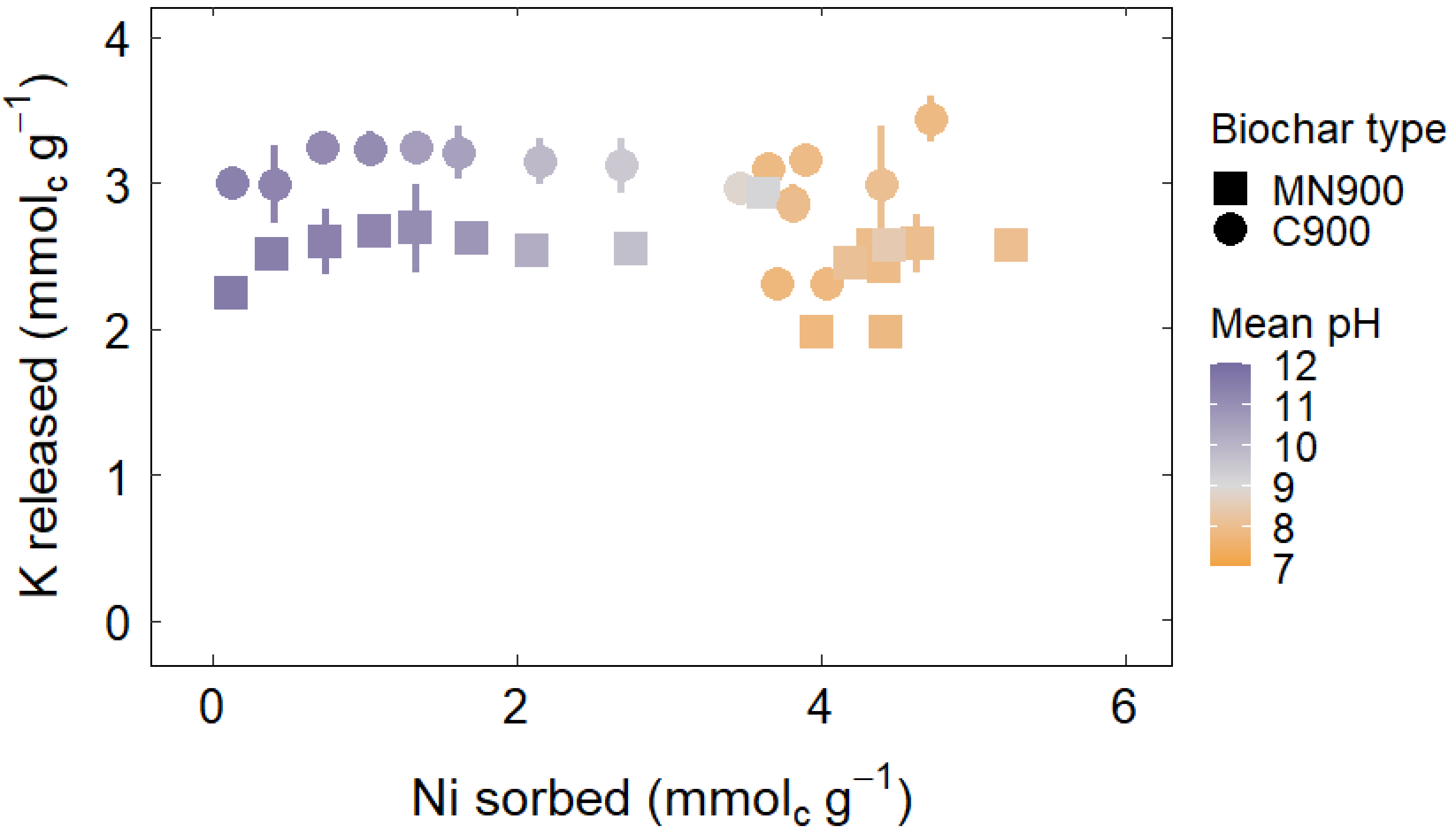


**Figure S7.** $K^+$ released by biochar into solution shown against Ni(II) removed from solution by biochar. $K^+$ ions are released from the biochar independently of the sorption of Ni(II) likely due to the high solubility of K-containing salts. The points are colored by final solution pH. When error bars representing ± one standard deviation are not visible, error is within the marker.

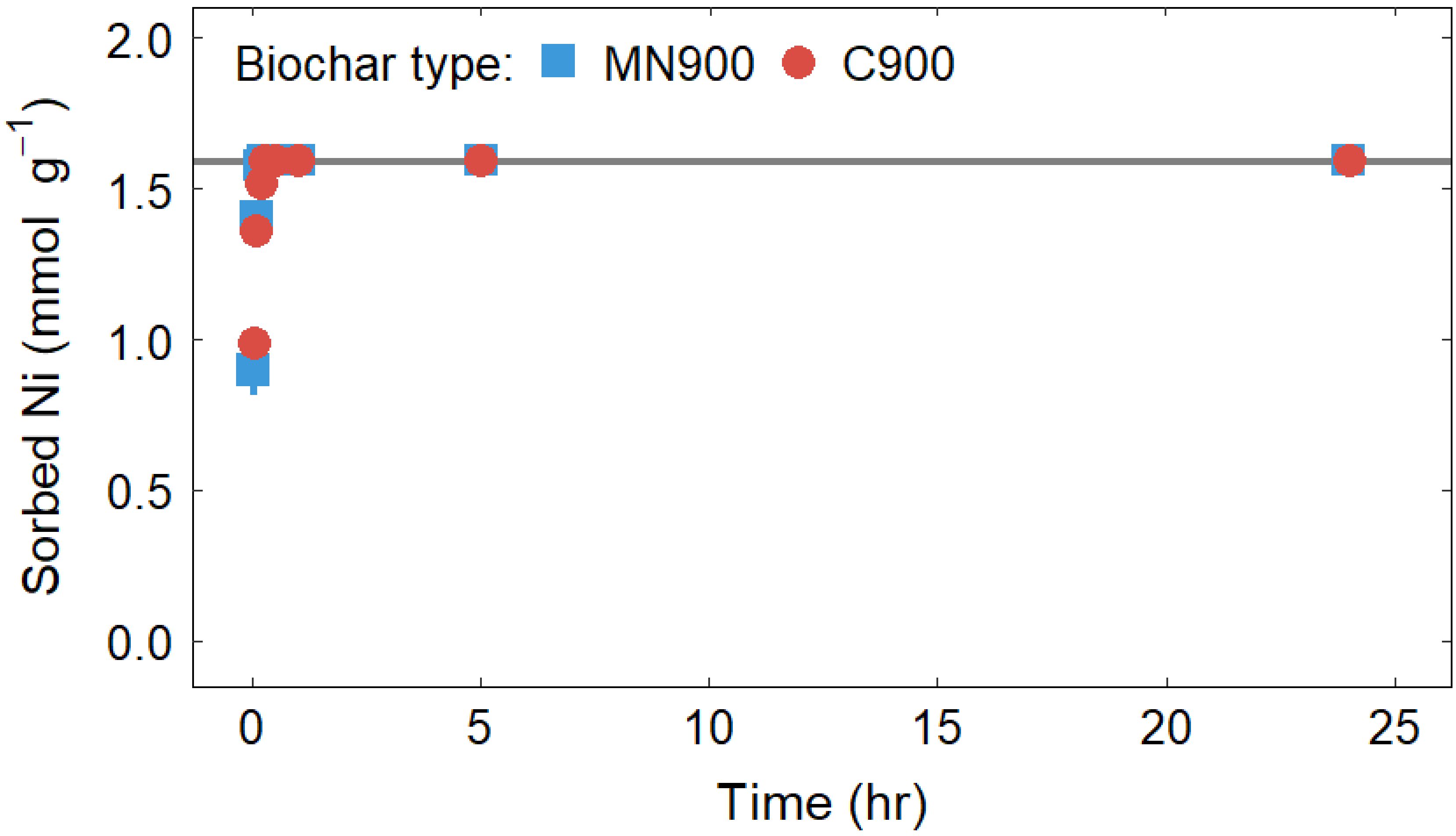


**Figure S8.** Ni(II) sorption kinetics over 24 hours with 5 mM Ni(II) and pH 5 in the initial solution. The gray line is the concentration of Ni(II) sorbed after 24 hours. When error bars representing ± one standard deviation are not visible, error is within the marker.

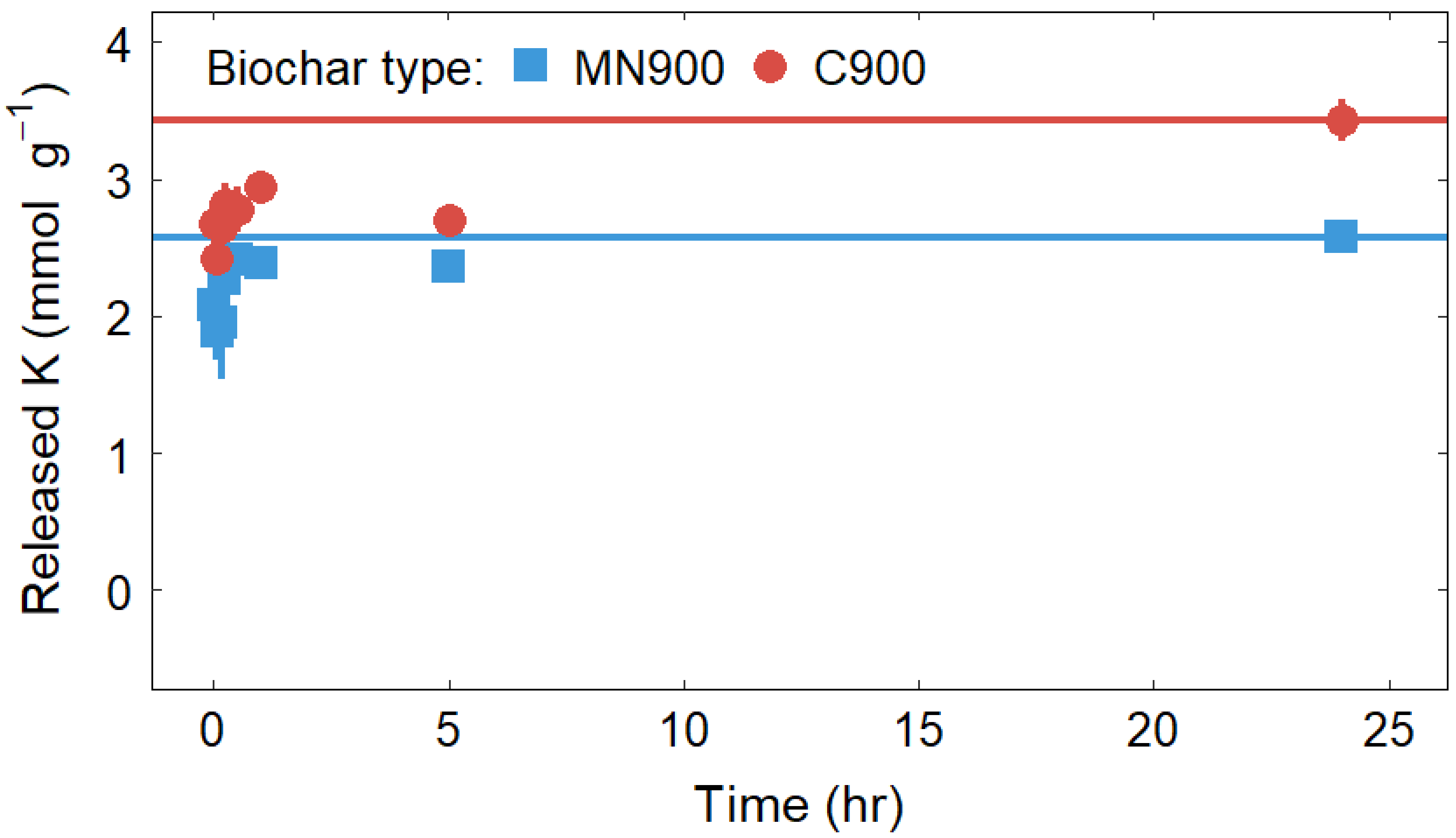


**Figure S9.** $K^+$ release kinetics over 24 hours with 5 mM Ni(II) and pH 5 in the initial solution. The colored lines are the concentration of $K^+$ released after 24 hours by C900 and MN900 from top to bottom, with the color matching the respective symbol colors. When error bars representing ± one standard deviation are not visible, error is within the marker.

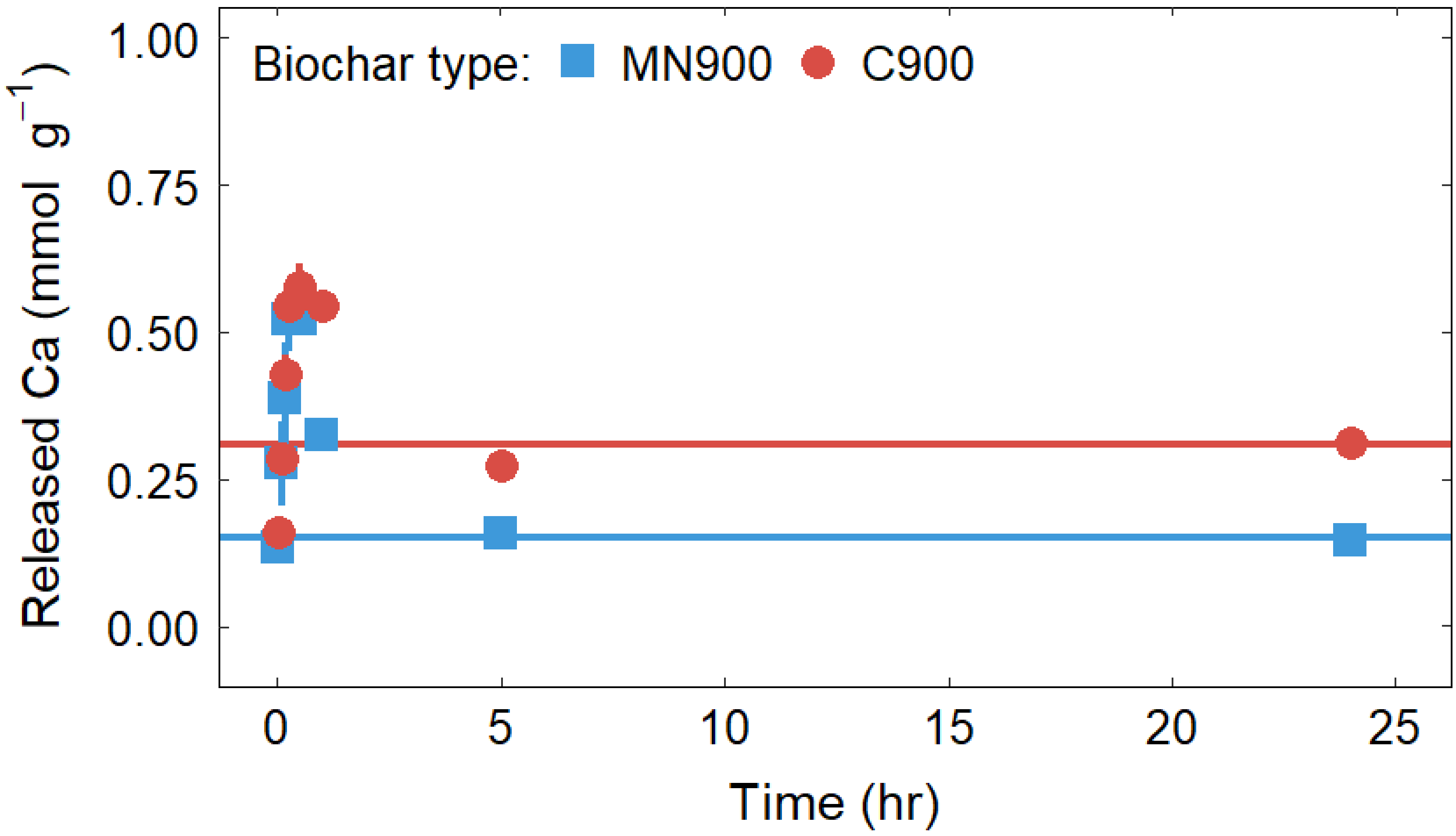


**Figure S10.** $Ca^{2+}$ release kinetics over 24 hours with 5 mM Ni(II) and pH 5 in the initial solution. The colored lines are the concentration of $Ca^{2+}$ released after 24 hours by C900 and MN900 from top to bottom, with the color matching the respective symbol colors. When error bars representing ± one standard deviation are not visible, error is within the marker.

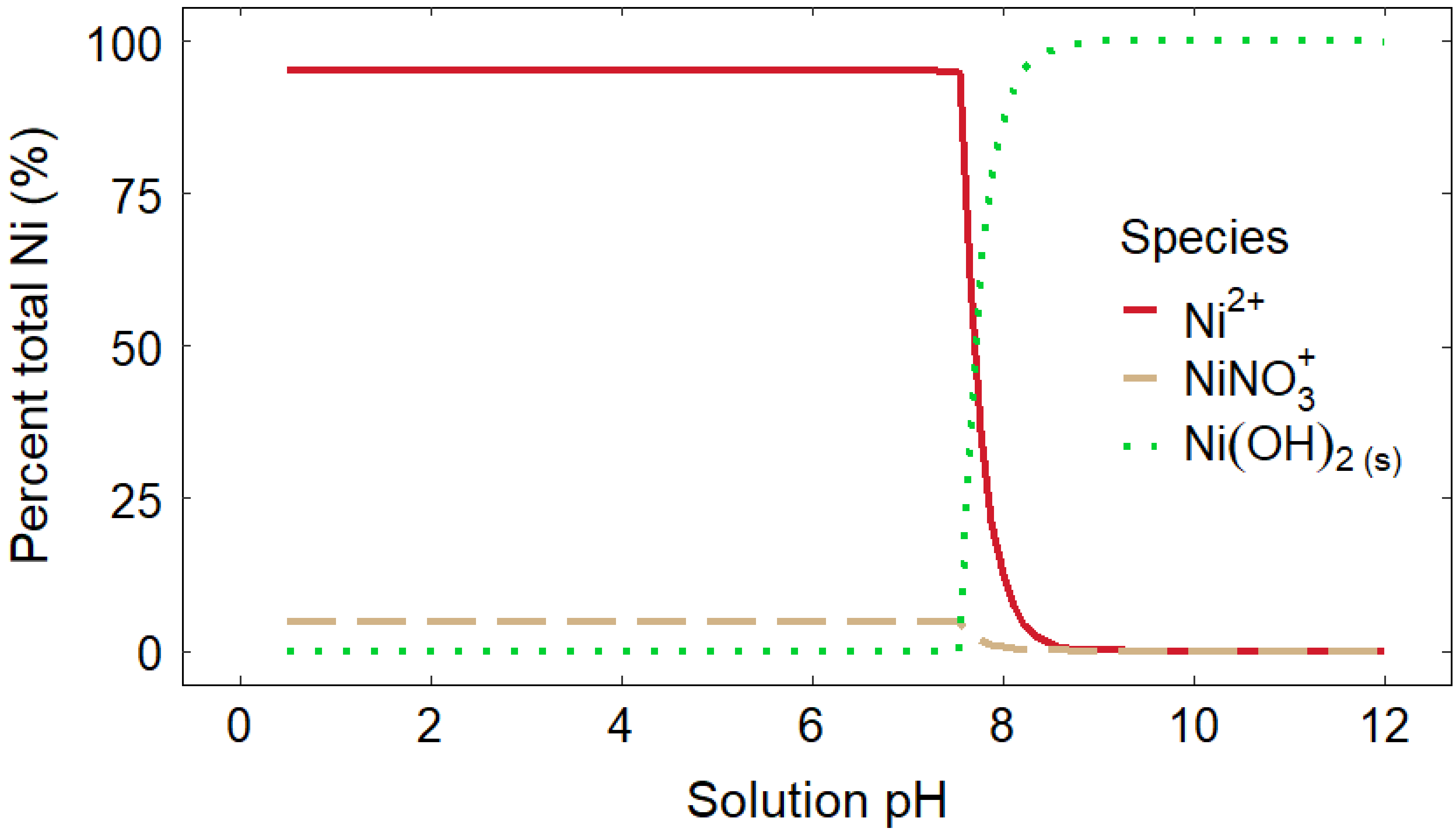


**Figure S11.** Ni speciation diagram in the 5 mM $Ni(NO_3)_2$, 10 mM $NaNO_3$ solution for pH values between 0.5 and 12. Calculations were completed using MINEQL+ 5.0 (Schecher and McAvoy, 1992).

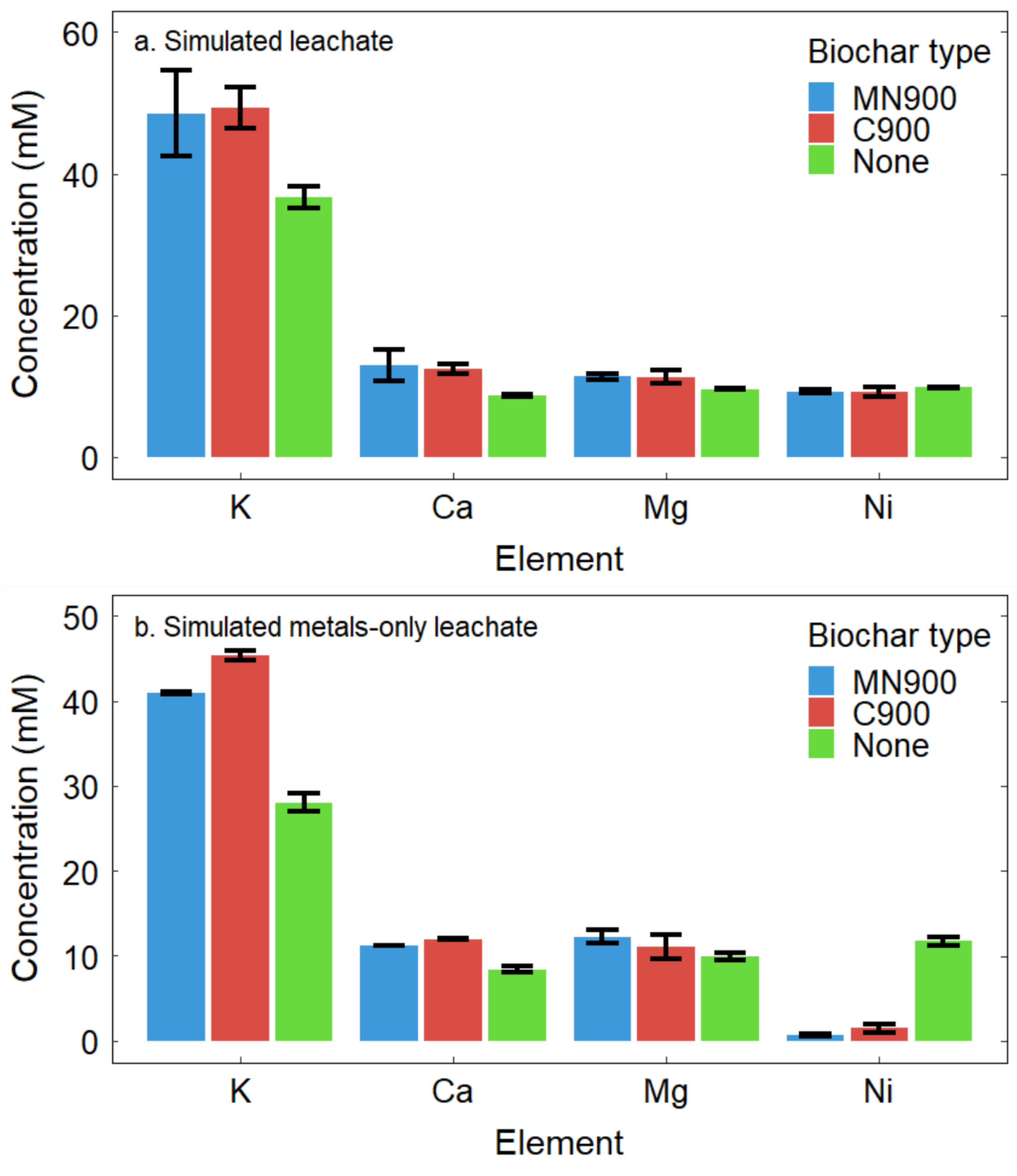


**Figure S12.** Elemental composition of solutions after 24 hours of contact between the indicated biochar and: a. simulated *O. chalcidica* leachate containing metals and organic acids and b. simulated *O. chalcidica* metals-only leachate solution. “None” refers to experimental controls with no biochar added. Error bars represent ± one standard deviation.

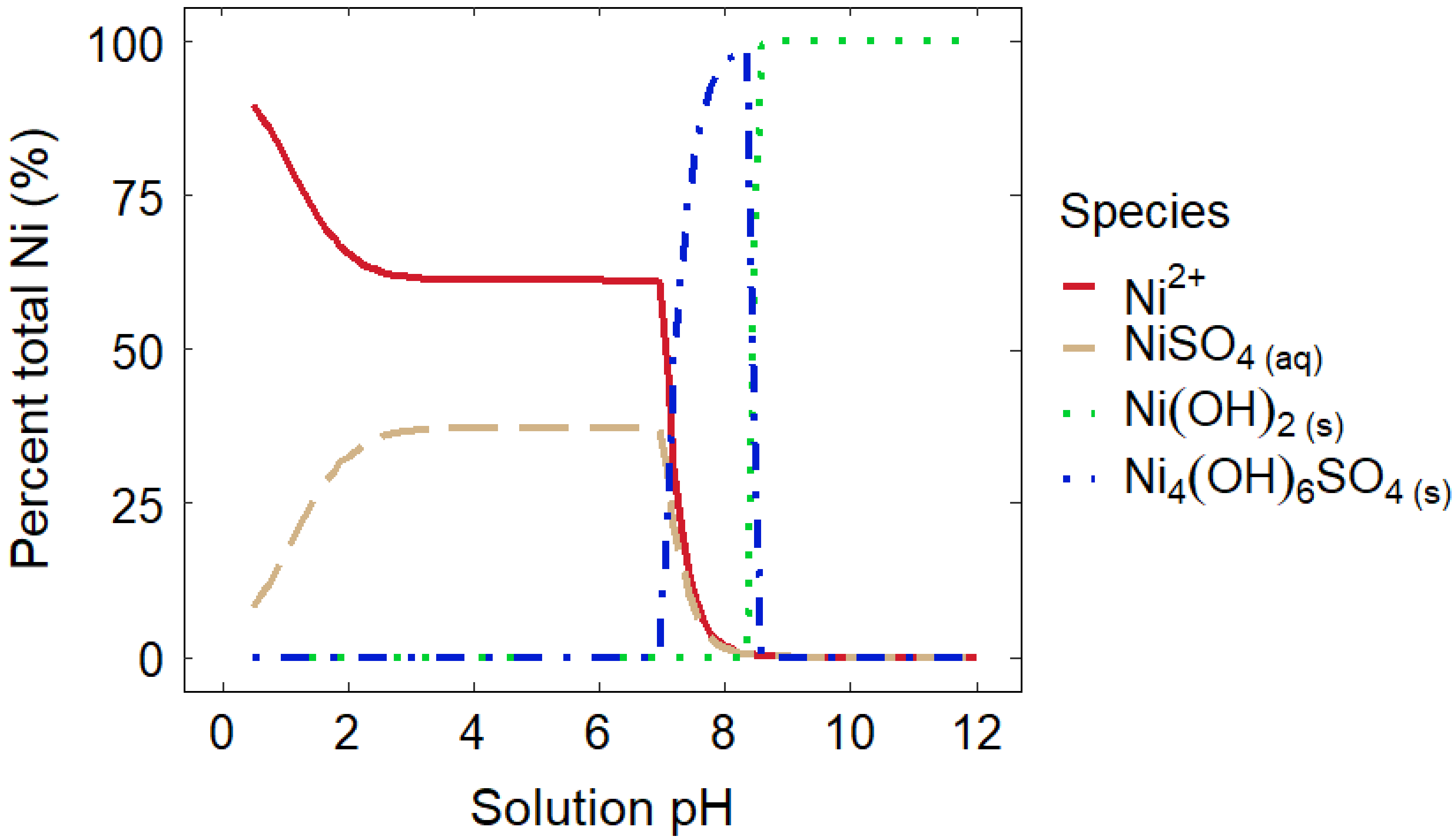


**Figure S13.** Ni speciation diagram in simulated Ni electroplating rinsewater solution with 0.2 g biochar for pH values between 0.5 and 12. Calculations were completed using MINEQL+ 5.0 (Schecher and McAvoy, 1992).

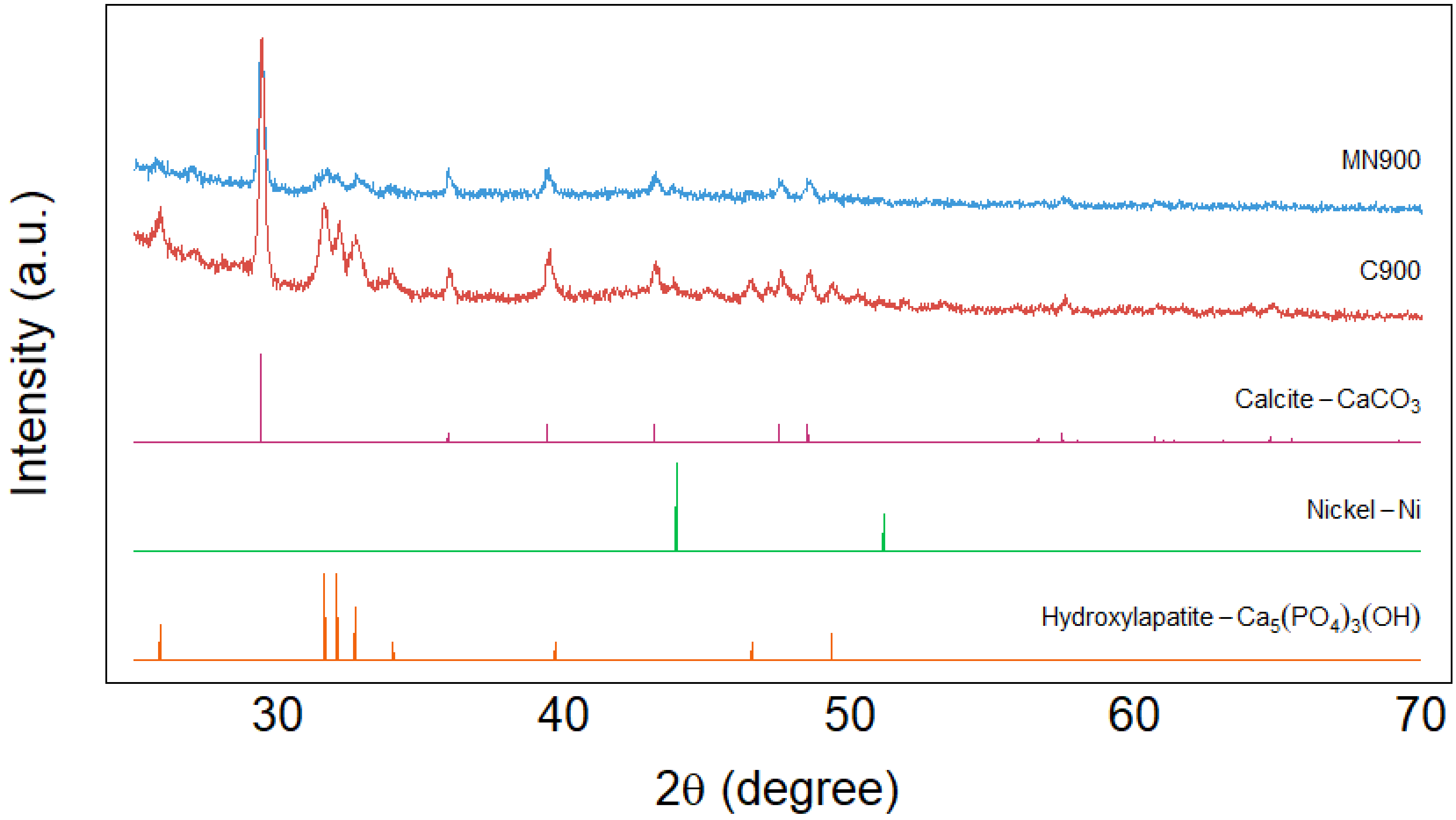


**Figure S14.** XRD spectra of *O. chalcidica* biochars after Ni5 sorption isotherm experiments and the relevant reference spectra calcite (PDF #99-000-0548), hydroxylapatite (PDF #99-000-1643), and Ni (PDF #01-077-3085). Hydroxylapatite and chlorapatite have similar compositions and lattice structures and are difficult to distinguish by XRD under measurement conditions in mixed-phase samples that may be subject to lattice strain or thermal annealing. EDS results show a spatial association between Ca, P, and Cl that indicate chlorapatite may be the appropriate phase identification.

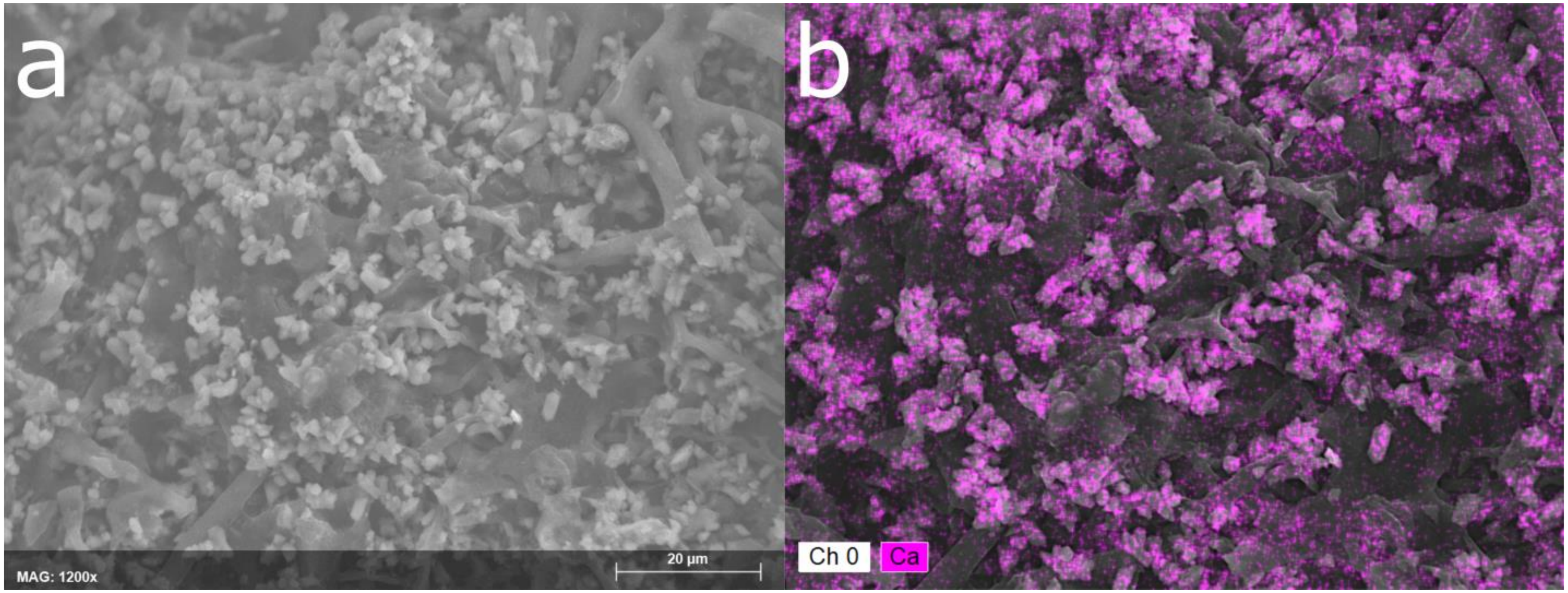


**Figure S15.** SEM-EDS of crystalline calcium structures on post-sorption MN900-Ni0 biochar with a. SEM image and b. the corresponding Ca EDS map. In b, Ch0 corresponds to channel 0, the grayscale image from the scanning electron microscope.

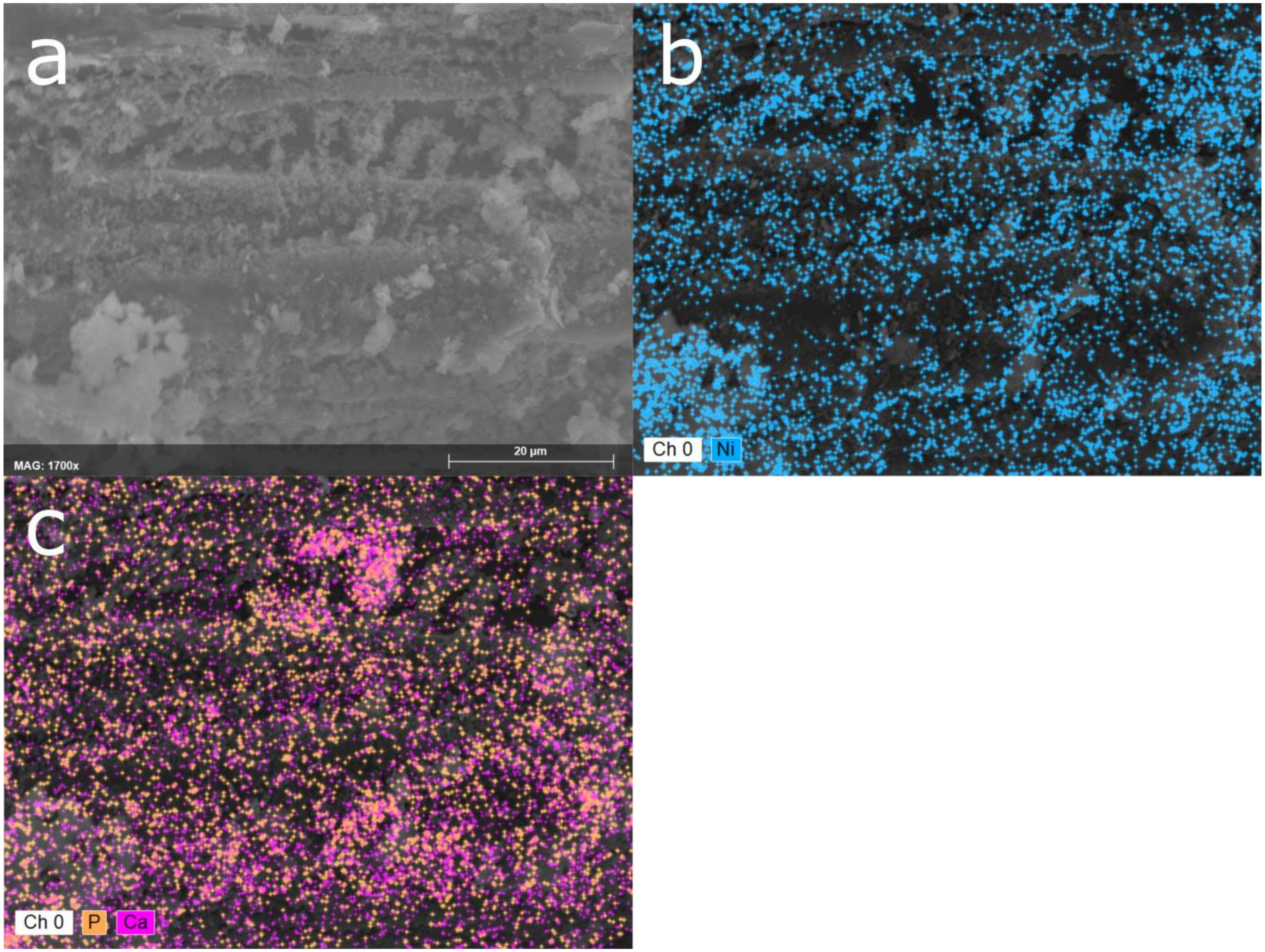


**Figure S16.** SEM-EDS of Ni, P, and Ca on post-sorption C900-Ni8 biochar with: a. SEM image, b. the corresponding Ni EDS map, and c. the corresponding Ca and P map. In b and c, Ch0 corresponds to channel 0, the grayscale image from the scanning electron microscope.

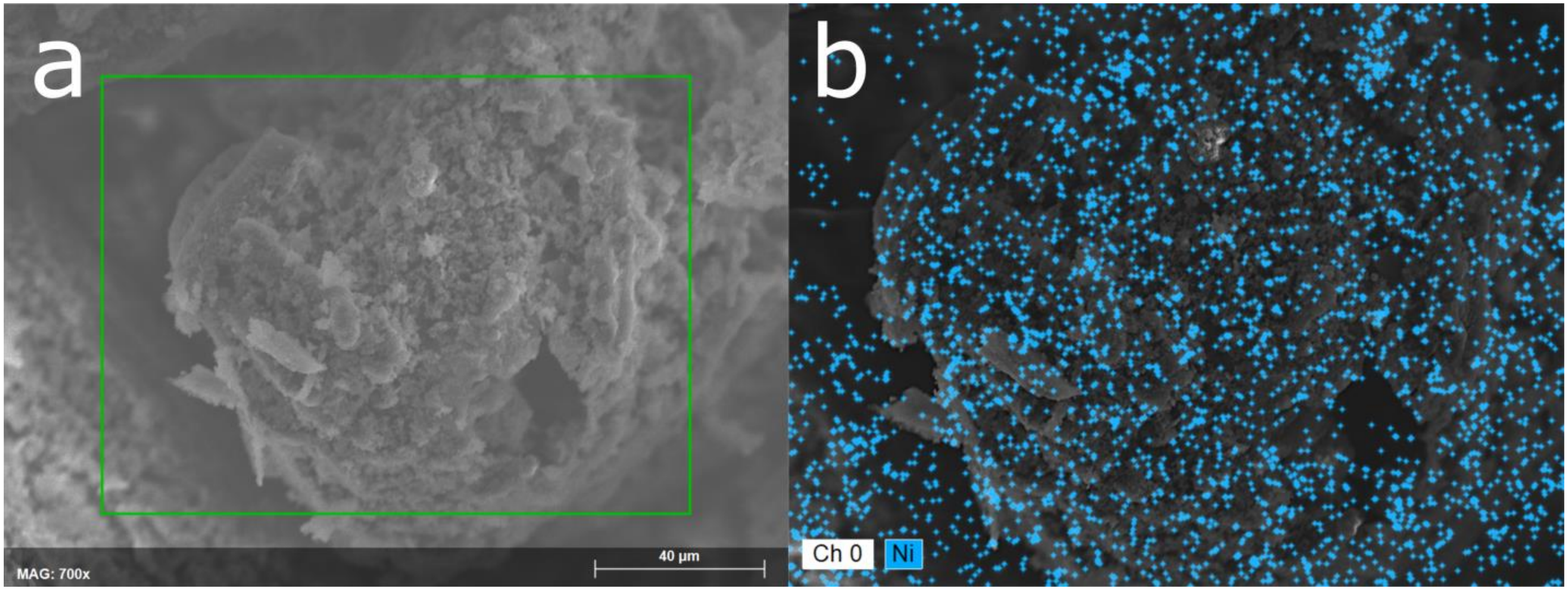


**Figure S17.** SEM-EDS of Ni on post-sorption MN900-Ni8 biochar with: a. SEM image and b. the corresponding Ni EDS map. In b, Ch0 corresponds to channel 0, the grayscale image from the scanning electron microscope. There is biochar in the background of the image.

## References


Benvenuti, T.; Krapf, R. S.; Rodrigues, M. A. S.; Bernardes, A. M.; Zoppas-Ferreira, J. Recovery of Nickel and Water from Nickel Electroplating Wastewater by Electrodialysis. *Sep. Purif. Technol.* **2014**, *129*, 106–112. https://doi.org/10.1016/j.seppur.2014.04.002.

Guilpain, M.; Laubie, B.; Zhang, X.; Morel, J.L.; Simonnot, M.-O. Speciation of nickel extracted from hyperaccumulator plants by water leaching. *Hydrometallurgy* **2018**, *180*, 192-200. 10.1016/j.hyrdomet.2018.07.024.

Schecher, W. D.; McAvoy, D. C. MINEQL+: A Software Environment for Chemical Equilibrium Modeling. *Comput. Environ. Urban Syst.* **1992**, *16*, 65–76. https://doi.org/10.1016/0198-9715(92)90053-T.
